# Cancer-associated adipocytes promote the invasion and metastasis in breast cancer through LIF/CXCLs positive feedback loop

**DOI:** 10.7150/ijbs.65227

**Published:** 2022-01-16

**Authors:** Chong Zhou, Xi He, Chang Tong, Honghui Li, Caifeng Xie, Yudong Wu, Lieliang Wang, Xiaohua Yan, Daya Luo, Yunpeng Tang, Zhongman Cheng, Xiangyang Xiong

**Affiliations:** 1 Department of Biochemistry and Molecular Biology, School of Basic Medical Sciences, Nanchang University, Nanchang, Jiangxi, 330006, China.; 2 Department of Breast Surgery, Jiangxi Provincial Cancer Hospital, Nanchang, Jiangxi, 330029, China.; 3 Pediatric Medical School, Nanchang University, Nanchang, Jiangxi, 330031, China.; 4 Second Clinical Medical School, Nanchang University, Nanchang, Jiangxi, 330031, China.; 5 School of Basic Medical Sciences, Nanchang University, Nanchang, Jiangxi, 330006, China.; 6 Province Key Laboratory of Tumor Pathogens and Molecular Pathology, Nanchang University, Nanchang, Jiangxi, 330006, China.; 7 Province Key Laboratory of Reproductive Physiology and Pathology, Nanchang University, Nanchang, Jiangxi, 330031, China.

**Keywords:** breast cancer, cancer-associated adipocyte, leukemia inhibitory factor, ELR+CXC Chemokines, invasion and metastasis

## Abstract

Cancer-associated adipocytes (CAAs), which are adipocytes transformed by cancer cells, are of great importance in promoting the progression of breast cancer. However, the underlying mechanisms involved in the crosstalk between cancer cells and adipocytes are still unknown. Here we report that CAAs and breast cancer cells communicate with each other by secreting the cytokines leukemia inhibitory factor (LIF) and C-X-C subfamily chemokines (CXCLs), respectively. LIF is a pro-inflammatory cytokine secreted by CAAs, which promotes migration and invasion of breast cancer cells via the Stat3 signaling pathway. The activation of Stat3 induced the secretion of glutamic acid-leucine-arginine (ELR) motif CXCLs (CXCL1, CXCL2, CXCL3 and CXCL8) in tumor cells. Interestingly, CXCLs in turn activated the ERK1/2/NF-κB/Stat3 signaling cascade to promote the expression of LIF in CAAs. In clinical breast cancer pathology samples, the up-regulation of LIF in paracancerous adipose tissue was positively correlated with the activation of Stat3 in breast cancer. Furthermore, we verified that adipocytes enhanced lung metastasis of breast cancer cells, and the combination of EC330 (targeting LIF) and SB225002 (targeting C-X-C motility chemokine receptor 2 (CXCR2)) significantly reduced lung metastasis of breast cancer cells *in vivo*. Our findings reveal that the interaction of adipocytes with breast cancer cells depends on a positive feedback loop between the cytokines LIF and CXCLs, which promotes breast cancer invasion and metastasis.

## Introduction

Breast cancer is one of the most common malignant tumors in females, with the highest mortality, and it is caused by uncontrolled proliferation of breast epithelial cells 1. Adipocytes account for a large proportion of human breast cancer tissues 2. In the early stage of breast cancer, cancer cells locally infiltrate the surrounding adipose tissue, which results in activation and transformation of adjacent adipocytes into cancer-associated adipocytes (CAAs) 3, 4. This confers advantages to breast cancer cells in survival, growth and metastasis 5. CAAs exhibit as dedifferentiation phenotype, which is mainly characterized by the down-regulation of adipocyte terminal differentiation marker gene expression such as peroxisome proliferator activated receptor gamma (PPAR-γ) and CCAAT enhancer binding protein alpha (C/EBP-α) 3. In addition, CAAs exhibit lipolysis, releasing metabolites that provide energy for tumors 6, 7. Importantly, compared with normal adipocytes, CAAs undergo inflammatory alterations and secrete more pro-inflammatory cytokines that can promote the growth and metastasis of breast cancer 2, 3.

The communication between adipocytes and cancer cells leads to adipocytes participating in breast cancer development, metastasis, and treatment resistance 8. Studies have shown that cancer cells over-express some pro-inflammatory factors, such as interleukin 6 (IL-6) and tumour necrosis factor α (TNF-α), are able to stimulate adipocytes to produce an activated phenotype in a paracrine fashion 9. Conversely, CAAs secrete chemokines and inflammatory cytokines such as C-C motif chemokine ligand 2 (CCL2), IL-6 and interleukin 1β (IL-1β) to promote cancer progress 10. Therefore, cytokines play an important role in the interaction between adipocytes and cancer cells.

Until now, the communication between adipocytes and cancer cells has not been completely elucidated. Therefore, an in-depth understanding of the crosstalk mechanism between CAAs and breast cancer cells is vital for exploring effective targets for breast cancer treatment. Here, we used the co-culture model of human adipocytes and breast cancer cells to explore the crosstalk of breast cancer cells and CAAs to figure out the underlying molecular mechanisms. We found that the pro-inflammatory cytokine LIF is a new mediator involved in the CAA-induced breast cancer invasion and metastasis. In addition, LIF and CXCLs can regulate mutually between breast cancer cells and CAAs, forming a positive feedback loop to promote the malignant development of breast cancer, while combining EC330 and SB225002 can significantly attenuate tumor metastasis. These findings provide new insights into the crosstalk between stromal cells and tumor cells in the microenvironment of breast tumors.

## Materials and methods

### Cytokines and neutralizing antibodies and inhibitors

Recombinant human LIF (#300-05, 20 ng/mL), CXCL3 (#300-40, 20 ng/mL) and CXCL8 (#200-08M, 20 ng/mL) were all purchased from Peprotech. LIF neutralizing antibody (AB-250-NA, 2 μg/mL) was purchased from R&D, and CXCL3 neutralizing antibody (PP1014P2, 5 μg/mL) was purchased from Origene. Stattic (#HY-13818), LY3214996 (#HY-101494), PD98059 (#HY-12028), SB 203580 (#HY-10256), LY294002 (#HY-10108), JSH-23 (#HY-13982), BAY 11-7082 (#HY-13453), T-5224 (#HY-12270), TK216 (#HY-122903), TAT-DEF-Elk-1 (#HY-P2262A), SB225002 (#HY-16711), Reparixin (#HY-15251), and EC330 (#HY-100949) were all purchased from Med Chem Express. SB-505124 (#M2250) was purchased from Abmole. A list of the concentrations and targets used for the inhibitors is provided in Supplementary Table 1.

### Antibodies

Antibodies against Stat3 (#9139; 1/1500 for western blot analysis, 1/800 for immunofluorescence), pY705-Stat3 (#9145; 1/1000 for western blot analysis, 1/100 for immunohistochemical staining), NF-κB p65 (#8242; 1/1000 for western blot analysis, 1/1600 for immunofluorescence), and pS536-NF-κB p65 (#3033; 1/1000) were purchased from Cell Signaling Technology; ERK1/2 (#AF0155; 1/1,000), and pT202/Y204-ERK1/2 (#AF1015; 1/1,000 for western blot analysis, 1/200 for immunofluorescence) were purchased from Affinity Biosciences. Antibodies against LIF (#ab2020; 1/200 for immunohistochemical staining) and CD44 (ab216647) were purchased from Abcam. CD90 (66766-1-Ig) and β-actin (#66009-1-Ig; 1/5000) were purchased from Proteintech.

### Patient specimens

According to the guidance of the local ethics committee, fat-rich human normal breast tissues and breast cancer tissues were obtained from breast cancer patients in Jiangxi Cancer Hospital through tumorectomy. All subjects provided their informed consent to participate in the study.

### Cell culture

Human triple-negative breast cancer (MDA-MB-231 and BT549 cells), estrogen receptor positive breast cancer (MCF-7 cells), human normal breast epithelial (MCF10A cells), mouse breast cancer (4T1 cells) and 3T3-L1 pre-adipocytes were purchased from the American Type Culture Collection (ATCC) or the Chinese National Infrastructure of Cell Line Resource. MDA-MB-231, MCF-7 and 3T3-L1 cells were maintained in DMEM (#10-013-CV, Corning) containing 10% FBS (#10099141C, Gibco), BT549 cells were maintained in RPMI Medium 1640 (#10-040-CV, Corning) containing 10% FBS, 4T1 were maintained in RPMI 1640 [+] L-glutamine (#99-595-CM, Corning) containing 10% FBS and MCF10A cells were maintained in MCF10A special medium (#CM-0525, Procell). All cells were cultured in a 5% CO_2_ cell incubator with a constant temperature of 37°C.

### Isolation and culture of primary human pre-adipocytes

Human primary pre-adipocytes were obtained from human adipose tissue of breast cancer mastectomy specimens. Briefly, the fresh adipose tissue was dissected carefully and minced. The adipose tissue pieces were digested with 1 mg/mL collagenase Ⅰ (#C8140, Solarbio) in a shaking incubator at 37 °C for 1 h. The digestion solution was filtered with a 100 µm cell sieve (#YZ-3479, Corning). After centrifugation, the upper liquid was discarded after centrifugation, and the lower stromal vascular fraction (SVF) was washed with PBS. Then the primary pre-adipocytes were maintained in DMEM/F12 (#31330095, Gibco) containing 10% FBS, as described by Liu L, et al. 11.

### Induced differentiation of pre-adipocytes

A differentiation-inducing medium containing 5 µg/mL insulin (#11061-68-0, Sigma), 0.5 mM IBMX (#28822-58-4, Sigma), 2 μM rosiglitazone (#122320-73-4, Sigma), and 0.5 μM dexamethasone (#50-02-2, Sigma) was applied to pre-adipocytes. The medium was changed every three days. After 15 days, the induction was complete. Then the cells were fixed with 4% paraformaldehyde for 15 min, Oil red O (#G1262, Solarbio) stained for 30 min, and then gently rinsed with PBS. The red-stained mature adipocytes and intracellular lipid droplets were observed under a phase-contrast microscope.

### Co-culture system and culture medium (CM) production

The Transwell chamber (#3415, Corning) was used as a co-culture medium for adipocytes and breast cancer cells. The adipocytes were seeded on the cell culture plate as the lower chamber. the upper frame was the Transwell chamber with a pore size of 0.4 μm, and the breast cancer cells were seeded in the upper chamber. After co-culturing for 24 h, the chamber was removed, and the medium of adipocyte was collected by cultivation in serum-free DMEM for another 24 h. After centrifugation, the supernatant was taken and stored at -80°C for later use.

### Hematoxylin and eosin (H&E) and immunohistochemistry (IHC)

Mammary tissues and breast cancer tissues were fixed with 4% formaldehyde for 4 h and transferred to 70% ethanol for 24 h, then embedded in paraffin wax and divided into 7 μm sections. After deparaffination, the sample sections were subsequently stained with hematoxylin and eosin.

For IHC, after deparaffinization and hydration, the tissue sections were incubated in 3% H_2_O_2_ for 10 min. The sections were washed twice with PBS and incubated in normal goat serum for 15 min. They were stained with anti-LIF and anti-p-Stat3 overnight at 4°C and then incubated with goat anti-rabbit IgG (#SA00001-2, Proteintech) for 1 h at room temperature. The peroxidase activity was visualized using a diaminobenzidine tetrahydrochloride (DAB) solution (#8059, Cell Signaling Technology). The sections were evaluated with an optical microscope (Olympus Optical). All patients signed informed consent for inclusion in the research project.

### Flow cytometry analysis

The pre-adipocytes were digested with trypsin, and 1 × 10^6^ cells were counted and fixed with 4% paraformaldehyde. After adding rupture fluid, the samples were centrifuged at 400 g for 5 min. Then, the supernatant was discarded, and the samples were incubated with 10-20 mg/mL of antibody in the dark for 45 min. After washing with PBS, cells were incubated with secondary fluorescently labeled IgG (#ab6854, Abcam) for 30 min. Then the cells were analyzed in a FACScan flow cytometer (Becton Dickinson).

### Wound-healing assay

Cells were inoculated in 12-well plates and fused to 100% the next day. Three parallel lines were drawn at equal distances in the wells where cells were cultured with a 200 μL pipette tip. Stimulating factors and 0.2% FBS were added to the DMEM medium, and then the migration of cells at the same location was observed every 12 h under a phase-contrast microscope, which was photographed and recorded.

### Transwell cell migration and Matrigel invasion assays

For the Transwell cell migration experiment, the cells in the logarithmic growth phase were digested to make a cell suspension, and 1 × 10^4^ cells were suspended in 200 μL of serum-free medium and seeded in the upper compartment of a 24-well Transwell chamber with a pore size of 8 μm (#3422, Corning). DMEM medium containing stimulating factors and 0.5% FBS was added to the lower chamber. After 24 h in a CO_2_ incubator, the uninvaded cells were discarded in the chamber. The invaded cells were fixed and stained with crystal violet for 20 min and rinsed 3 times with ddH_2_O. After the chamber was dried, the number of cells at the bottom of the chamber was observed in bright field mode. Five visual fields were taken randomly and the number of cells was calculated.

For the Matrigel invasion experiment, Matrigel (#354234, Corning) was spread on the Transwell chamber. The remaining steps were same as above.

### MTT assay

In 96-well plates, 5 × 10^4^ cells per well were seeded, and DMEM medium containing stimulating factors was added to incubated each sample for 48 h. Then, 20 μL of MTT (#M1020, Solarbio) was added to each well and the samples were left for 4 h at 37°C. Then, the DMSO was added to each well at room temperature. After 30 min, the absorbance at 450 nm was measured in a SpectraMax® Paradigm® microplate reader (Molecular Devices).

### Enzyme linked immunosorbent assay (ELISA)

According to the manufacturer's protocol, LIF, CXCL1, CXCL2, CXCL3, and CXCL8 were measured with ELISA kits (#EK1107, #EK196, #EK1264, #EK1265, and #EK108, MultiSciences). For the standard solution or sample 100 μL/well was added to the plate containing the specific enzyme-linked antibody and and the sample was incubated at room temperature for 2 h. After washing, 100 μL/well of horseradish peroxidase-labeled streptavidin was added to it and the sample was incubated at room temperature for 45 min. And 100 μL of luminescent substrate TMB was added to each well, and then the sample was incubated at room temperature for 15 min. Finally, the reaction was terminated. A SpectraMax® Paradigm® microplate reader (Molecular Devices) was used to detect the absorbance at 450 nm and 570 nm.

### Small interfering RNA (siRNA) transfection

After the cells were fused to 60%, replaced the complete medium with new DMEM. The Lipofectamine™ 2000 (#11668027, Thermo Fisher) was diluted with DMEM and left it at room temperature for 5 min. The siRNA (Customized from RiboBio) was diluted with DMEM to a final concentration of 50 nM. Then, the Lipofectamine™ 2000 and siRNA solution were mixed and left it at room temperature for 15 min. the mixture was added to the cells, and transfected cells for 6 h and the samples were collected on the third day to detect the transfection efficiency or perform follow-up experiments. The siRNA sequences are listed below:

Stat3 #1—5'- AGACCCGTCAACAAATTAA-3';

Stat3 #2—5'- CATCGAGCAGCTGACTACA-3'.

### Western blots

For immunoblotting analysis, cells were lysed on ice in lysis buffer (50 mM Tris-HCl, pH 7.5, 150 mM NaCl, 0.5% NP40, 1 mM EDTA, 10 mM NaF, 10 mM Na_4_P_2_O_7_, and 1 mM Na_3_VO_4_). Then the lysate was centrifuged at 4°C. The protein sample was diluted with protein loading buffer and denatured at 98°C. The same amounts of the protein samples were loaded on the SDS-PAGE, and electrophoresis was run at 80 V. The protein was transferred to nitrocellulose at 200 mA. Bovine serum albumin (5%) was used to block the immunoblots, and it was incubated with the specific antibody at 4°C overnight, and then with secondary antibody at 4°C for 4 h. HPR was used as a substrate to detect the immunoblots.

### Total RNA extraction and real-time quantitative PCR

RNA isolation was performed using Trizol (#T9108, TaKaRa) according to the manufacturer's instructions. To purify RNA from breast tissues, 100 mg of the tissue samples was cut up, 1 mL of Trizol was added and the tissue was ground through a homogenizer to extract tissue RNA. Reverse transcription of 1 μg total RNA was performed with the ReverTra Ace®q-PCR RT kit (#FSQ-101, TOYOBO) according to the manufacturer's instructions. Then real-time quantitative PCR (q-PCR) was performed using the SYBR® Premix Ex TaqTM Kit (#DRR820A, TaKaRa) on a Step One Plus Real-Time PCR system (Applied Biosystems). Relative Gene expression was determined after normalization to GAPDH for breast cancer cells or S18 for adipocytes/CAAs and calculated with the following formula: relative expression level ¼2 ddCT.

Primer sequences are listed in Supplementary Table 2.

### Transcriptome sequencing (RNA-seq) analysis

The total RNA of the sample was extracted and enriched with Oligo (dT) beads. The mRNA was fragmented with fragmentation buffer, and random primers were used to reverse transcribed into cDNA. The second strand cDNA was synthesized by DNA polymerase I, RNase H, dNTP, and buffer. Then, the QiaQuick PCR extraction kit was used to purify the cDNA fragment, poly (A) was added, and the Illumina sequencing adapter was connected. The ligation products were selected by agarose gel electrophoresis, PCR amplified, and transcriptome sequencing was performed using the Illumina HiSeq TM 2500 sequencer (Berry Genomics).

### Immunofluorescence

The adipocytes were fixed in 4% paraformaldehyde for 15 min. Then, 0.5% Triton X-100 was used to permeabilize the cells for 10 min, and 5% bovine serum albumin was used to block the cells for 30 min. The samples were incubated with the specific antibody overnight, and then with the corresponding species of fluorescent-conjugated secondary antibody (#E-AB-1055, Elabscience) for 30 min using DAPI (#AR1176, BOSTER). The staining was detected under a laser scanning confocal microscope (FV3000, Olympus).

### Tail vein metastasis assays

SPF-grade Balb/c mice (six-week-old females) were purchased from Nanchang University (Nanchang, Jiangxi, China). All experiments described were approved by the Animal Research Ethics Committee of Nanchang University.

3T3-L1 cells were induced to differentiate into mature adipocytes. Then, 4T1 cells and 3T3-L1 cells were co-cultured, and EC330 (1 μM) and SB225002 (0.1 μM) were added alone or in combination for 3 days. After treatment, the 4T1 cells (1 × 10^6^ cells per mouse) were injected intravenously in mice (n=6 per group). Tumors were allowed to grow for 2 weeks. Mice were executed and lung tissues were taken for analyses. All lung tissues were paraffin-embedded in 10% formaldehyde fixation and stained with H&E, and metastatic nodules were further confirmed microscopically and measured by ImageJ software program 12, 13.

### Statistical analysis

Mean ± S.D. was used to present experimental quantitative data. The Student's t-test was performed for statistical analysis of the Matrigel invasion assay, ELISA results, and quantitative PCR (q-PCR) results. ***p < 0.001, **p < 0.01 and * p < 0.05. Error bars are ± S.D.

## Results

### Cancer-associated adipocytes promote breast cancer cell migration and invasion

To explore how adipocytes affect breast cancer cells in the tumour microenvironment, we isolated primary pre-adipocytes from breast cancer mastectomy specimens. The positive expression rates of pre-adipocyte markers CD44 and CD90 were more than 90% by flow cytometry, which were consistent with the surface marker expression characteristics of adipocytes (Supplementary Figure 1A) 14. Then, the pre-adipocytes were treated with differentiation-inducing medium for 15 days. The q-PCR results showed that compared with pre-adipocytes, mature adipocytes up-regulated PPAR-γ, C/EBP-α and fatty acid binding protein 4 (FABP4), while hormone-sensitive lipase (HSL) and pre-adipocyte factor 1 (PREF1) expression were down-regulated (Supplementary Figure 1B), showing that pre-adipocytes were induced to differentiate into mature adipocytes.

Then, we co-cultured pre-adipocytes and adipocytes with breast cancer cells respectively. Due to the pore size of the Transwell chamber used (0.4 μm), the adipocytes have no direct contact with breast cancer cells, but crosstalk still occurs between them through soluble factors in the co-culture model. After co-culturing for 24 h, the expression of PPAR-γ and C/EBP-α in adipocytes was down-regulated, HSL expression was up-regulated, and the expression of pro-inflammatory cytokines IL-1β, IL-6 and CCL2 increased significantly (Supplementary Figure 1C) compared with the adipocytes without co-culturing treatment. We noticed that these gene levels were less altered in co-cultured pre-adipocytes than in co-cultured mature adipocytes (Supplementary Figure 1C and D). These results suggest that breast cancer cells have little effect on pre-adipocytes. In addition, the Oil red O staining showed that the lipid droplets in the adipocytes were densely arranged, while the lipid droplets in the co-cultured adipocytes were loosely distributed and smaller in volume (Supplementary Figure 1E). The above results indicate that after co-culturing with breast cancer cells, the adipocytes are transformed into CAAs.

Next, the culture medium of adipocytes was acted on breast cancer cells. The migratory capacity of MDA-MB-231 cells treated with conditioned medium from CAA (CAA-CM) increased significantly, whereas the migratory capacity of cells treated with conditioned medium for mature adipocytes (Adi-CM) did not changed significantly (Supplementary Figure 1F). Similarly, CAA-CM effectively promoted the invasion of MDA-MB-231 and BT549 cells, while the cell invasion ability of the Adi-CM treatment group was not significantly improved (Supplementary Figure 1G). These results indicate that CAAs can significantly promote breast cancer cells migration and invasion, while adipocytes have little effects on breast cancer.

### LIF is highly expressed in breast cancer-associated adipocytes

**Figure 1:**
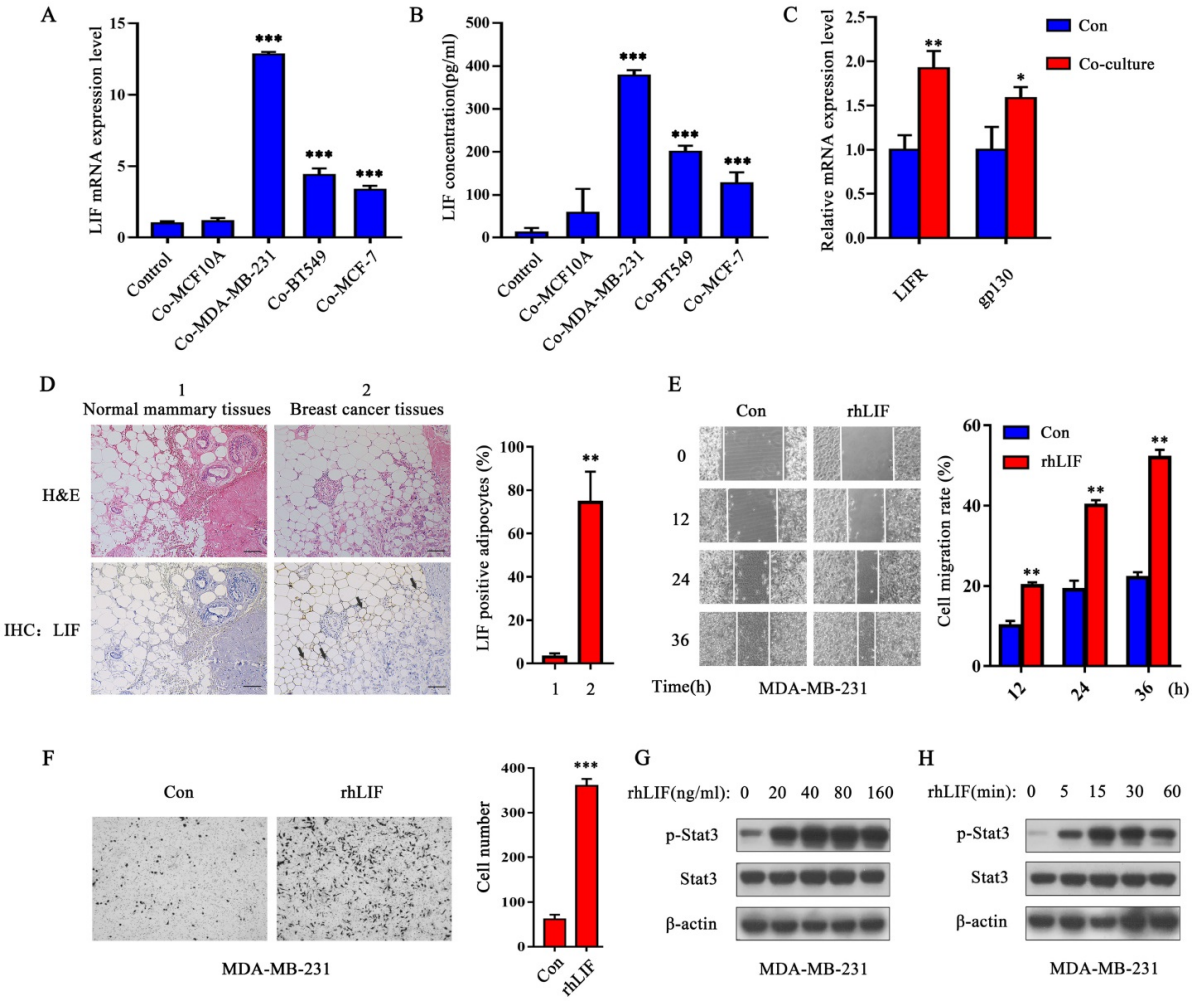
**LIF promotes breast cancer cell migration and invasion by activating Stat3 signaling.** (**A**) After co-culturing with breast epithelial cells or breast cancer cells for 24 h, the LIF mRNA expression of adipocytes in each group was detected by q-PCR. (**B**) After co-culturing, the breast epithelial cells or breast cancer cells in the upper chamber were removed, adipocytes were further cultured with serum-free DMEM for another 24 h, and the LIF in the medium was quantified by ELISA. (**C**) Breast cancer MDA-MD-231 cells were co-cultured with adipocytes for 24 h, q-PCR detected the expression of LIFR and gp130 mRNA in breast cancer cells. (**D**) IHC detected the expression of LIF in normal breast tissue and breast cancer tissue, the highly expressed LIF is pointed by the black arrow. The corresponding quantization chart is shown on the right, scale bar: 200 μm. (**E and F**) MDA-MD-231 cells were treated with rhLIF and subjected to migration (**E**) and Transwell Matrigel invasion (**F**) assays, the corresponding quantization chart is shown on the right. (**G** and **H**) MDA-MD-231 cells were treated with 0, 20, 40, 80, 160 ng/mL rhLIF for 15 min and were treated with rhLIF for different time (0, 5, 15, 30, 60 min), the Stat3 phosphorylation was analyzed by western blot. Typical microscopic fields and blots are shown and quantitative data are presented as mean ± SD from at least three independent experiments. *p < 0.05, **p < 0.01, ***p < 0.001.

In the RNA-Seq data of the previous study, we found that the cytokine LIF was differentially expressed between adipocytes and CAAs 11, which implied that LIF has a potential impact on the microenvironment of breast cancer. Therefore, we detected the expression and secretion of LIF in co-cultured pre-adipocytes and muture adipocytes. The mRNA level of LIF increased in both pre-adipocytes and mature adipocytes after co-culturing for 24 h and the expression of LIF was higher in co-cultured mature adipocytes (Supplementary Figure 2A and B). Moreover, increased protein expression and secretion levels of LIF were only detected in co-cultured mature adipocytes (Supplementary Figure 2C and D). MDA-MB-231 cells secreted less LIF, as well as no significant increase in LIF secretion after co-culturing with adipocytes (Supplementary Figure 2D). The above results indicate that LIF is mainly secreted by CAAs. Therefore, we focused on exploring the role of CAA-derived LIF in breast cancer progression. By co-culturing adipocytes with several breast cancer cell lines, the q-PCR results showed that the LIF expression of adipocytes co-cultured with human normal breast epithelial MCF-10A cells did not change compared with the control group, but the expression of LIF mRNA was significantly up-regulated in adipocytes co-cultured with breast cancer MDA-MB-231, BT549 and MCF-7 cells compared with the control group (Figure 1A). Similarly, the ELISA results showed that the level of LIF secreted by adipocytes increased significantly, especially in the co-culture group with triple-negative breast cancer MDA-MB-231 and BT549 cell lines. In addition, the adipocytes co-cultured with MCF10A cells secreted less LIF proteins (not statistically significant compared with the control group), while the adipocytes in the control group almost did not secret LIF (Figure 1B). Then we evaluated the response of breast cancer cells to LIF signals in the co-culture system. LIFR and gp130 are receptor complexes that are necessary to receive LIF signals 15. The q-PCR results showed that the expression of LIFR and gp130 increased in the co-cultured MDA-MB-231 cells (Figure 1C). This desmonstrates that breast cancer cells induce CAAs to highly express LIF and secrete LIF protein.

Next, we performed H&E staining and IHC analysis on the pathological tissue sections of breast cancer patients (Supplementary Table 3). The results showed that breast cancer cells invaded adipose tissue and caused the morphology of adipocytes to gradually become smaller and irregular (Figure 1D), which means that they are transformed by breast malignancies. The expression of LIF in adipocytes adjacent to cancer cells were increased significantly (as the black arrow points out), while the adipocytes in normal breast tissue did not express LIF (Figure 1D). These results indicate that breast cancer-related adipocytes highly express LIF which may act on neighboring breast cancer cells in a paracrine manner to promote breast cancer progression.

### The rhLIF promotes breast cancer cell migration and invasion by activating the Stat3 pathway

Human recombinant LIF (rhLIF) protein was used to evaluate the effect of LIF on breast cancer cells *in vitro*. The migration and invasion capabilities of breast cancer cells were detected by wound healing and Transwell Matrigel invasion assays. The results showed that rhLIF (20 ng/mL) markedly promoted the migration (Figure 1E and Supplementary Figure 2E) and invasion ability of MDA-MB-231 cells (Figure 1F) and BT549 cells (Supplementary Figure 2F). The MTT results showed that rhLIF had no obvious effect on the proliferation of breast cancer cells (Supplementary Figure 2G). The above results suggest that LIF affects the motility of breast cancer cells rather than their proliferation.

The Stat3 signaling pathway is found to be abnormally activated in a variety of malignant tumors 16, 17, and Stat3 is one of the main signaling pathways downstream targets of LIF 18, 19. We considered it a necessity to explore whether LIF can regulate the migration and invasion of breast cancer cells through activation of the Stat3 signaling pathway. The western blot results showed that rhLIF can significantly enhance Stat3 phosphorylation, and the phosphorylation level of Stat3 in MDA-MB-231 and BT549 cells was the strongest after rhLIF treatment for 15 min (Figure 1G and H, and Supplementary Figure 2H). Therefore, LIF can promote breast cancer migration and invasion, and the Stat3 signal is likely to participate in this process.

### LIF neutralizing antibody reverses the effect of CAA on breast cancer cell migration and invasion

**Figure 2:**
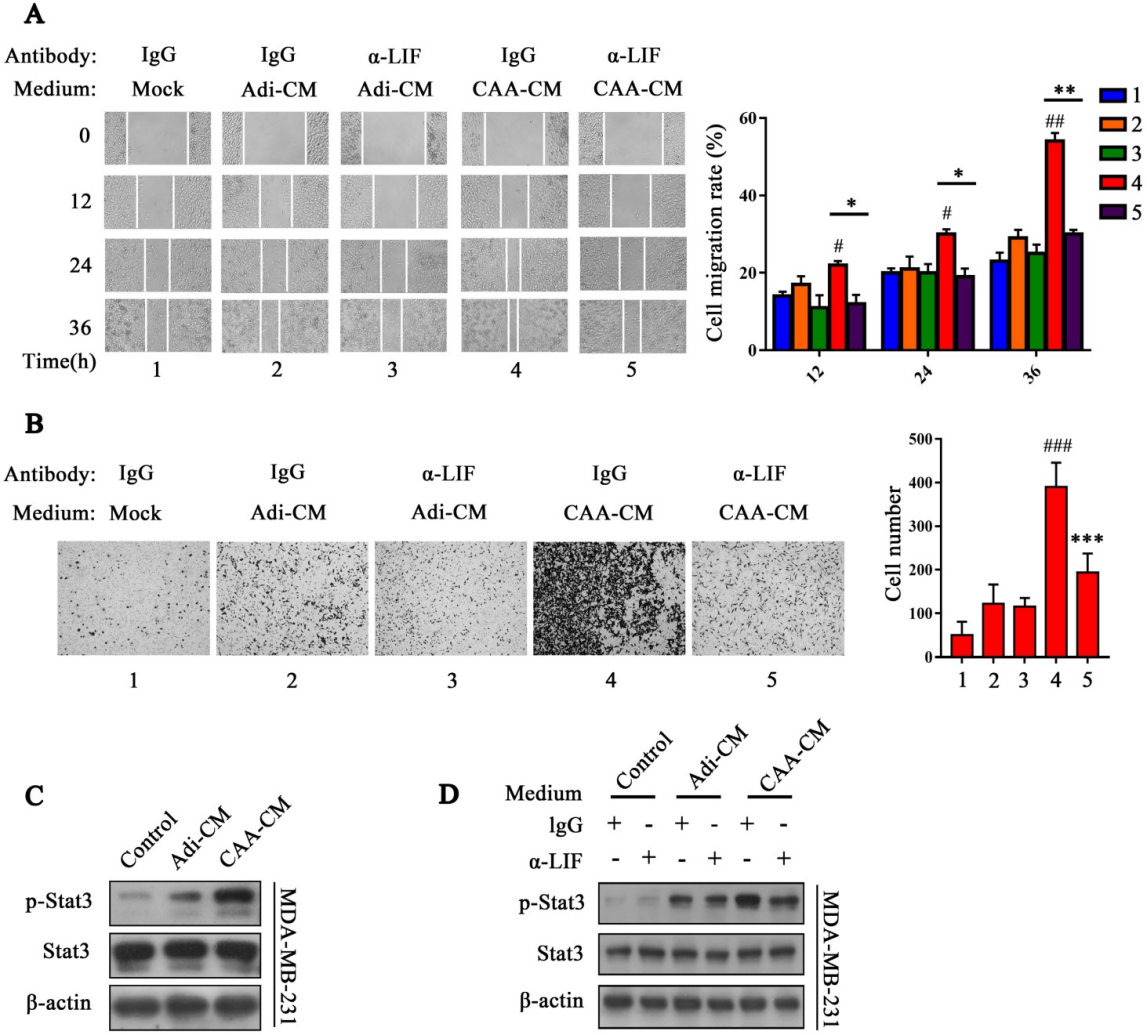
**LIF neutralizing antibody inhibits the migration and invasion capability and the Stat3 phosphorylation induced by CAA-CM on breast cancer cells. (A** and **B)** MDA-MD-231 cells cultured in Adi-CM or CAA-CM or control DMEM were treated with LIF neutralizing antibody/IgG. The cell migration (**A**) and invasion (**B**) situation were monitored under a phase contrast microscope. The corresponding quantization chart is shown on the right. (**C**) MDA-MB-231 cells were treated with different culture media for 15 min, and Stat3 phosphorylation was analyzed by western blot. (**D**) MDA-MD-231 cells cultured in DMEM or Adi-CM or CAA-CM were treated with LIF neutralizing antibody or IgG for 15 min, and Stat3 phosphorylation was analyzed by western blot. Typical microscopic fields and blots are shown and quantitative data are presented as mean ± SD from at least three independent experiments. ^#^*p* < 0.05, ^##^*p* < 0.01, ^###^*p* < 0.001, CAA-CM VS. Mock; *p < 0.05, **p < 0.01, ***p < 0.001, CAA-CM + α-LIF VS. CAA-CM.

In the tumor microenvironment, LIF has multiple functions, such as promoting cancer cell metastasis, immunosuppression, and tumor angiogenesis, as well as even negatively regulating tumor progression 20, 21. Different sources of LIF have different effects on cancer cells. To further explore whether CAA-derived LIF also exerts this cancer-promoting effect, LIF-neutralizing antibody (α-LIF) was used to neutralize LIF in CAA-CM. We found that LIF neutralizing antibody (2 μg/mL) greatly reduced the effect of CAA-CM on the migration and invasion of MDA-MB-231 (Figure 2A and B) and BT549 (Supplementary Figure 2I and 2J) cells.

Next, we tested the activation of the Stat3 signal in MDA-MB-231 and BT549 cells by adding the adipocyte culture supernatant of each group. The results showed that Stat3 phosphorylation of MDA-MB-231 cells triggered by CAA-CM was significantly increased compared with Adi-CM and control cells (Figure 2C), and co-cultured adipocytes showed significantly increased Stat3 phosphorylation than co-cultured pre-adipocytes (Supplementary Figure 2K). Furthermore, the LIF neutralizing antibody (2 μg/mL) can inhibit Stat3 phosphorylation of MDA-MB-231 and BT549 cells induced by CAA-CM (Figure 2D and Supplementary Figure 2L). These results further indicate that CAA-derived LIF is likely to exert a cancer-promoting effect via the Stat3 signal.

### Stat3 mediates CAA-derived LIF to induce breast cancer cell migration and invasion

**Figure 3:**
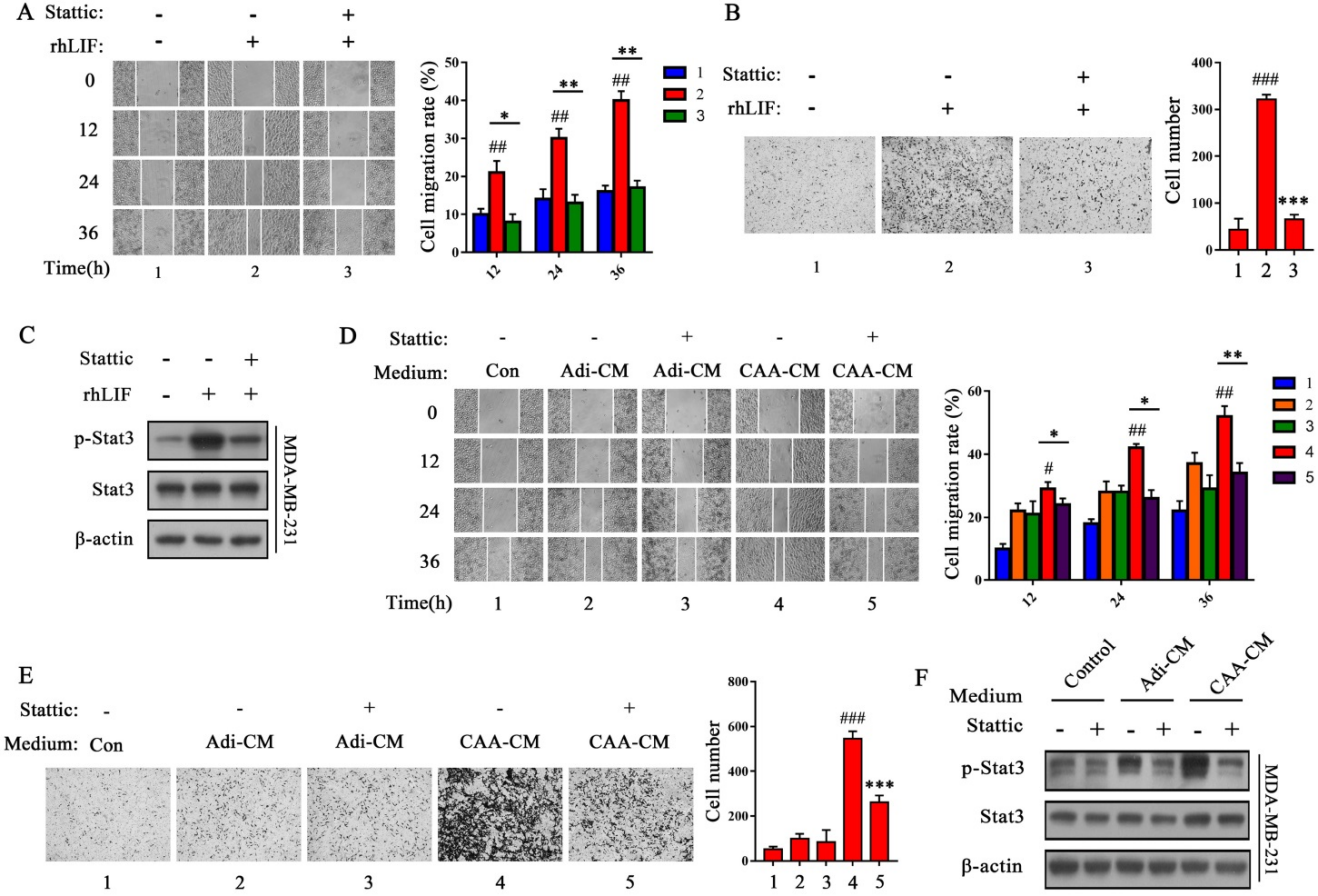
** Stattic inhibits the migration and invasion of MDA-MB-231 cells and Stat3 phosphorylation induced by rhLIF and CAA-CM.** (**A** and **B**) MDA-MD-231 cells were stimulated with rhLIF or DMEM with Stattic and subjected to migration (**A**) and Transwell Matrigel invasion (**B**) assays. The corresponding quantization chart is shown on the right. (**C**) MDA-MD-231 cells were stimulated by rhLIF combined with Stattic for 15 min, and Stat3 phosphorylation was analyzed by western blot. (**D** and **E**) MDA-MD-231 cells were stimulated by DMEM, Adi-CM or CAA-CM combined with Stattic and subjected to migration (**D**) and Transwell Matrigel invasion (**E**) assays. The corresponding quantization chart is shown on the right. (**F**) MDA-MD-231 cells were treated with DMEM or different adipocyte culture medium and Stattic for 15 min, and Stat3 phosphorylation was analyzed by western blot. Typical microscopic fields and blots are shown and quantitative data are presented as mean ± SD from at least three independent experiments. ^#^*p* < 0.05, ^##^*p* < 0.01, ^###^*p* < 0.001, rhLIF or CAA-CM VS. Con; *p < 0.05, **p < 0.01, ***p < 0.001, rhLIF + Stattic VS. rhLIF or CAA-CM + Stattic VS. CAA-CM.

**Figure 4:**
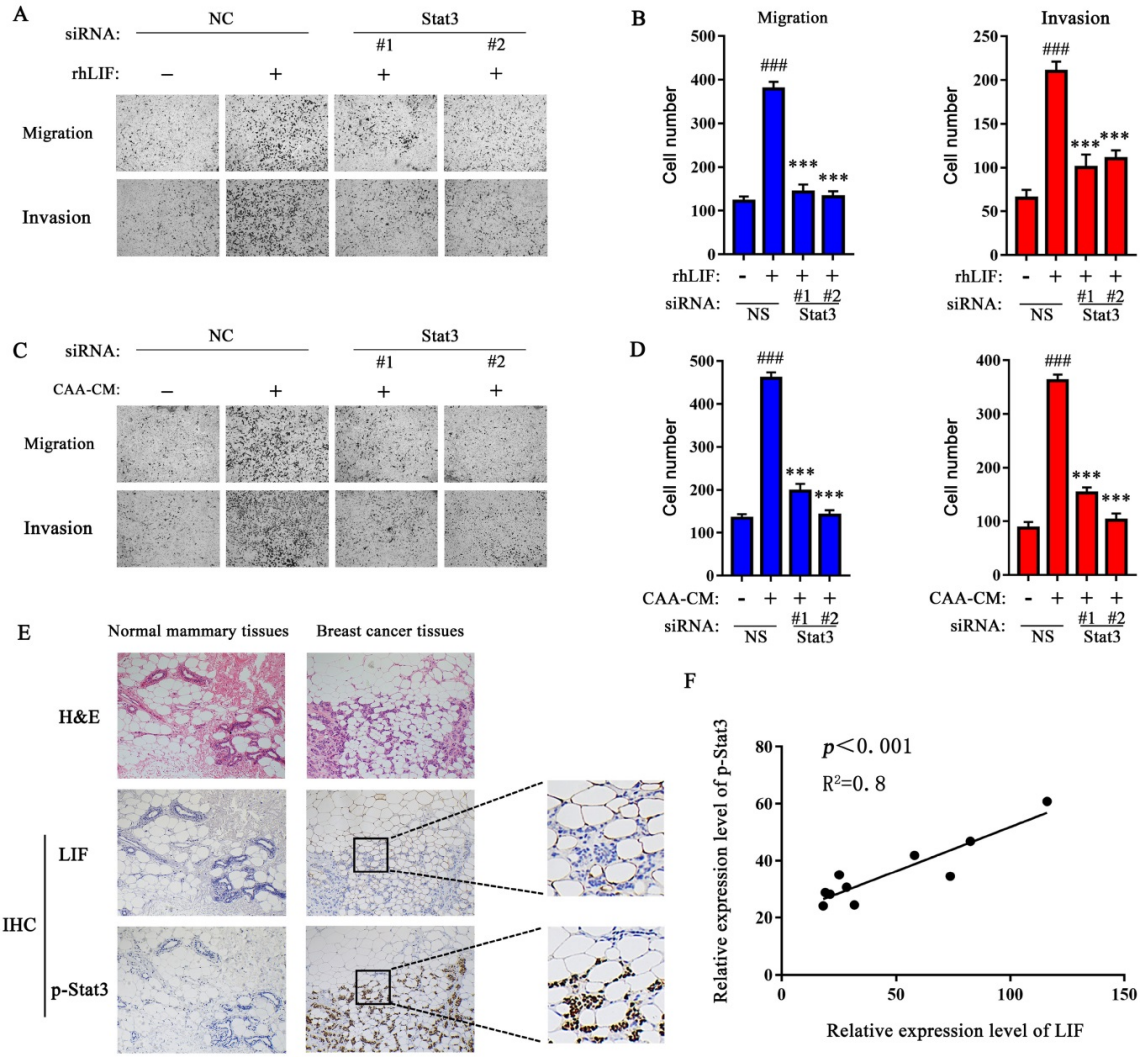
** Knockdown of endogenous Stat3 with siRNA inhibits the migration and invasion of breast cancer cells induced by CAA-CM and rhLIF.** (**A**-**D**) MDA-MB-231 cells transfected with siRNA were treated with rhLIF (**A** and **B**) or CAA-CM (**C** and **D**) to observe cell migration and invasion. (**E**) The expression of LIF and Stat3 phosphorylation in normal breast tissue and breast cancer tissue was analyzed by IHC, scale bar: 100 μm. (**F**) The correlation between the relative expression of LIF and Stat3 phosphorylation in human breast cancer sections. Typical microscopic fields and blots are shown and quantitative data are presented as mean ± SD from at least three independent experiments. ^#^*p* < 0.05, ^##^*p* < 0.01, ^###^*p* < 0.001, NS + rhLIF or CAA-CM VS. NS; *p < 0.05, **p < 0.01, ***p < 0.001, siRNA+rhLIF or CAA-CM VS. NS + rhLIF or CAA-CM.

To evaluate whether Stat3 mediates CAA-derived LIF to induce breast cancer cells migration and invasion, we examined the effects of Stattic, a specific Stat3 inhibitor, that inhibits Stat3 phosphorylation 22. We found that Stattic (10 μM) greatly rescued the rhLIF-induced migration and invasion of breast cancer MDA-MB-231 (Figure 3A and B) and BT549 cells (Supplementary Figure 3A and B), and inhibited rhLIF-induced Stat3 phosphorylation in MDA-MB-231 (Figure 3C) and BT549 cells (Supplementary Figure 3C). Stattic also weakened the effect of CAA-CM on MDA-MB-231 and BT549 cell migration (Figure 3D and Supplementary Figure 3D), and invasion (Figure 3E and Supplementary Figure 3E). Our results indicate that CAAs can regulate the migration and invasion of breast cancer cells via the LIF/Stat3 axis. In addition, Stattic inhibited CAA-CM induced phosphorylation of Stat3 in breast cancer MDA-MB-231 (Figure 3F) and BT549 cells (Supplementary Figure 3F). Furthermore, two siRNAs (Stat3 #1 and Stat3 #2) were used to knock down the endogenous Stat3 expression in MDA-MB-231 cells to further verify our inferences. The results showed that both siRNA fragments significantly knocked down the expression of the Stat3 protein (Supplementary Figure 3G). Then, the Transwell migration and Matrigel invasion assays showed that the knocking down of endogenous Stat3 can reverse the promotion of rhLIF and CAA-CM on breast cancer cell migrationand invasion (Figure 4A, B, C and D). Therefore, these results indicate that the Stat3 signal in breast cancer cells is a key signal molecule for CAAs to promote the migration and invasion of triple-negative breast cancer cells.

### The expression of LIF in adipocytes adjacent to breast cancer is positively correlated with Stat3 phosphorylation in breast cancer tissues

Next, IHC was used to evaluate the correlation between the expression of LIF in adipocytes adjacent to breast cancer and Stat3 phosphorylation in breast cancer tissues. Compared with normal breast tissue, Stat3 phosphorylation was positively expressed in the nucleocytoplasmic region of cancer cells (Figure 4E), and its relative expression showed a positive correlation with the LIF in adipocytes adjacent to breast cancer (Figure 4F), while the LIF and Stat3 phosphorylation levels in normal breast tissues were negative. These results confirmed that the Stat3 signal of breast cancer cells was significantly activated and positively correlated with the high expression of LIF in adjacent adipose tissue.

### ERK1/2 activates transcription factors Stat3 and NF-κB to up-regulate the expression of LIF in CAAs

**Figure 5:**
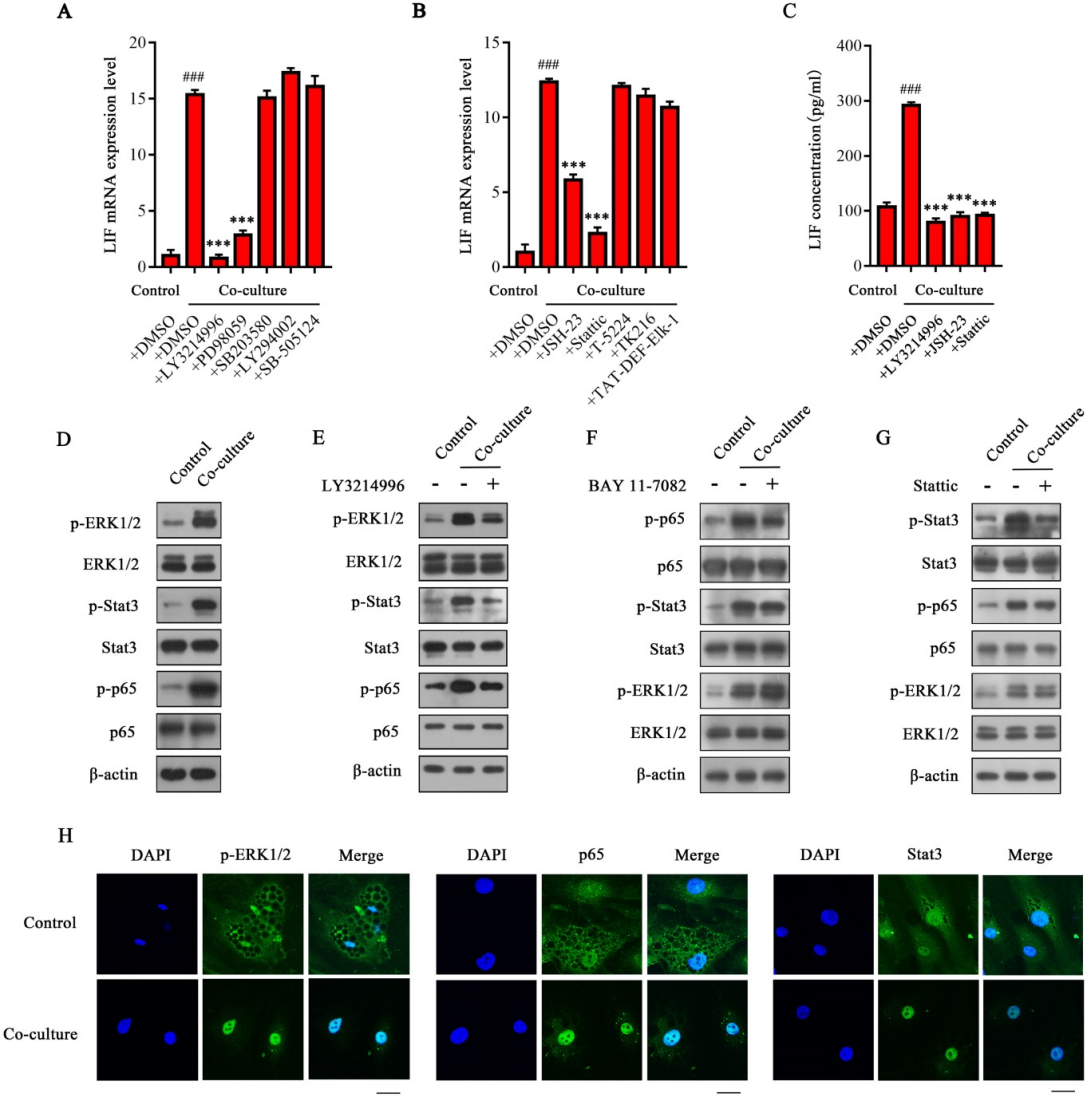
**ERK1/2 up-regulates LIF expression by activating NF-κB and Stat3 signals in CAA.** (**A** and **B**) MDA-MB-231 cells and adipocytes were co-cultured in the Transwell system, and selective pathway inhibitor (**A**) or ERK1/2 downstream transcription factor inhibitor were added to detect expression (**B**) and secretion (**C**) of LIF. (**D**) The protein expression level in adipocytes was analyzed by western blot. (**E**-**G**) LY3214996 (**E**), BAY 11-7082 (**F**), or Stattic (**G**) were added to the co-culture system of MDA-MB-231 cells and adipocytes. The protein expression level in adipocytes was analyzed by western blot. (**H**) The adipocytes were labeled with specific antibodies of p-ERK1/2, NF-κB p65 and Stat3, and the expression and localization of the three in adipocytes were observed under a fluorescent confocal microscope, scale bar: 20 μm. Typical microscopic fields and blots are shown and quantitative data are presented as the mean ± SD from at least three independent experiments. ^#^*p* < 0.05, ^##^*p* < 0.01, ^###^*p* < 0.001, Co-culture + DMSO VS. Control + DMSO; *p < 0.05, **p < 0.01, ***p < 0.001, Co-culture +inhibitors VS. Co-culture + DMSO.

To elucidate the molecular mechanism underlying the high expression of LIF in CAAs, specific inhibitors of the signaling pathway that are closely related to both the function and expression of LIF were used to screen the upstream signaling pathway that regulates the expression of LIF in CAAs. The results showed that the ERK1/2 specific inhibitor LY3214996 could completely inhibit the expression of LIF mRNA in CAAs. The MEK selective inhibitor PD98059 also significantly inhibited the expression of LIF mRNA in CAAs. In contrast, the p38 specific inhibitor SB203580, PI3K specific inhibitor LY294002 and TGF-β specific inhibitor SB-505124 had no apparent inhibitory effect on the expression of LIF (Figure 5A), indicating that the expression of LIF in CAAs may be regulated by the ERK1/2 signal. Considering that the ERK1/2 downstream transcription factors mainly include c-Fos, ETS, ELK-1, Stat3, NF-κB, etc. 23-26, these transcription factors may be involved in the regulation of the ERK1/2 signal on LIF. As shown in Figure 5B, only the NF-κB inhibitor JSH-23 and the Stat3 inhibitor Stattic treatment group significantly downregulated the LIF mRNA levels in CAAs. The c-Fos inhibitor T5224, ETS inhibitor TK216 and ELK-1 inhibitor TAT-DEF-ELK-1 had no noticeable effect on the expression of LIF. The ELISA results showed that LY3214996, JSH-23, and Stattic reduced the content of LIF in the CAA-CM (Figure 5C). Based on the above experimental results, we speculate that after co-culturing with breast cancer cells, the ERK1/2 signaling pathway in CAAs is activated, which results in further activation of transcription factors Stat3 and NF-κB to initiate LIF expression.

To confirm the hypothesis above, we evaluated the activation of related signal pathways in CAAs. The phosphorylation levels of ERK1/2, Stat3 and NF-κB p65 were significantly increased in CAA after co-culturing with MDA-MB-231 cells (Figure 5D). indicating that ERK1/2, Stat3, and NF-κB signaling pathways in CAAs were all activated. The specific inhibitor LY3214996 not only inhibited the activation of ERK1/2, but also inhibited the phosphorylation of Stat3 and NF-κB p65 (Figure 5E) in CAAs, indicating that ERK1/2 can further activate the Stat3 and NF -κB signal. Furthermore, BAY 11-7082 (inhibiting the binding of the p65 subunit of NF-κB to DNA) significantly inhibited the phosphorylation of the p65 protein without affecting the phosphorylation levels of ERK1/2 and Stat3 (Figure 5F). Similarly, Stattic did not affect ERK1/2 and p65 phosphorylation but only inhibited the Stat3 phosphorylation of its corresponding target protein (Figure 5G). These results indicate that ERK1/2 is the upstream signal molecule of Stat3 or NF-κB, and Stat3 and NF-κB signals are independent of each other, so they do not affect each other's regulation in CAAs. However, the phosphorylation levels of ERK1/2 were not altered in co-cultured or control pre-adipocytes (Supplementary Figure 3H), suggesting that after co-culturing, the ERK1/2 signaling pathway mainly functions in the interactions between mature adipocytes and breast cancer cells. In addition, the immunofluorescence results showed that the phosphorylation level of ERK1/2 in CAAs was significantly enhanced, and the expression of Stat3 and p65 in the nucleus was significantly increased (Figure 5H). In summary, we confirmed that the ERK1/2 signaling pathway is activated in CAAs and then regulates the activation of downstream transcription factors Stat3 and NF-κB (p65) to initiate LIF expression.

### CXCLs are highly expressed in breast cancer cells co-cultured with adipocytes

**Figure 6:**
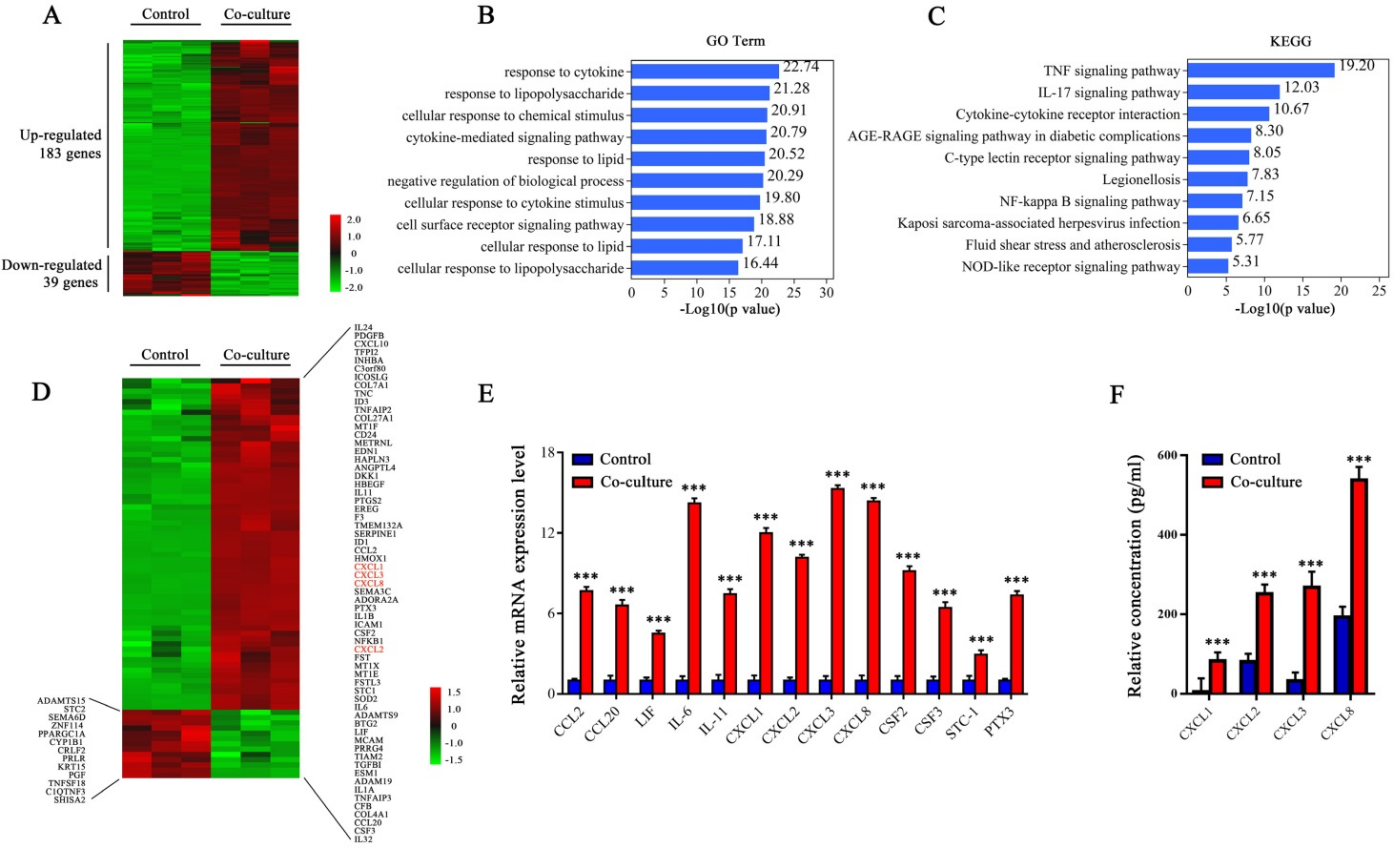
** Transcriptome and secretome profiling of MDA-MB-231 cells by RNA-Seq.** (**A**) The heat map of 222 differentially expressed genes selected from the control and co-cultured MDA-MB-231 cells. The color correspond to the relative gene expression level and is represented by Log2 Fold Change. (**B** and **C**) GO analysis (**B**) and KEGG pathway enrichment (**C**) of the 222 differentially expressed genes. The top 10 GO terms or pathways are presented. (**D**) Heat map of genes differentially expressing secretory proteins between the control and co-cultured MDA-MB-231 cells. The color correspond to the relative gene expression level as the Log2 Fold Change. (**E**) The expression levels of differentially secretory protein genes were detected by q-PCR. (**F**) The secretion levels of CXCLS were detected by ELISA. Quantitative data are presented as mean ± SD from at least three independent experiments. *p < 0.05, **p < 0.01, ***p < 0.001, Co-culture VS. Control.

Previously, we found high expression of LIF in CAA but not in adipocytes, indicating that this phenomenon may be caused by breast cancer. Therefore, we believe that it is the key factor that activates the ERK1/2 signal and up-regulates LIF expression released by breast cancer cells. Next, RNA-seq was used to screen secreted proteins of breast cancer cells that induce CAAs to express LIF. MDA-MB-231 cells cultured alone were used as the control group (control), and MDA-MB-231 cells co-cultured with adipocytes were used as the experimental group (co-culture), with three parallel samples in each group. The sequencing results retain genes with Log2 Fold Change ≥ 1.0, Log2 Fold Change ≤ -1.0, and false discovery rate (FDR) < 0.05. There were 183 genes up-regulated and 39 genes down-regulated in the co-cultured MDA-MB-231 cells (Figure 6A). Then, we performed the Gene Ontology (GO) term and Kyoto Encyclopedia of Genes and Genomes (KEGG) enrichment analysis on these differentially expressed genes. Through GO Term analysis, it was found that the lipolysis reaction, cytokines, and related signal pathways were highly enriched in co-cultured MDA-MB-231 cells (Figure 6B). Through KEGG analysis, it was found that some significantly up-regulated signal transduction pathways, including the TNF-α signal, IL-17 signal, chemokine receptor and NF-κB signal, which are closely related to inflammation, cell migration and invasion (Figure 6C). We further analyzed the genes of secreted proteins in the differentially expressed genes and found that 63 genes were up-regulated, and 13 genes were down-regulated in the co-cultured MDA-MB-231 cells. The expressions of CXCLs, including CXCL1, CXCL2, CXCL3 and CXCL8, which are members of the ELR+CXC chemokine subfamily, were significantly increased in co-cultured MDA-MB-231 cells (Figure 6D).

Next, we verified the mRNA levels of some differentially expressed genes, including CXCL1, CXCL2, CXCL3, and CXCL8, in the control and co-cultured MDA-MB-231 cells. Consistent with the RNA-seq results, co-cultured MDA-MB-231 cells highly expressed CXCL1, CXCL2, CXCL3, and CXCL8 (Figure 6E) , and the secretion levels of CXCL1, CXCL2, CXCL3, and CXCL8 were significantly increased (Figure 6F). Therefore, we believe that breast cancer cells express more CXCL1, CXCL2, CXCL3, and CXCL8 after co-culturing with adipocytes. These chemokines may be the key molecules that activate the ERK1/2 signaling pathway and up-regulate the expression of LIF in CAAs.

### The receptor CXCR2 is involved in activating ERK1/2 signals to up-regulate LIF expression in CAAs

**Figure 7:**
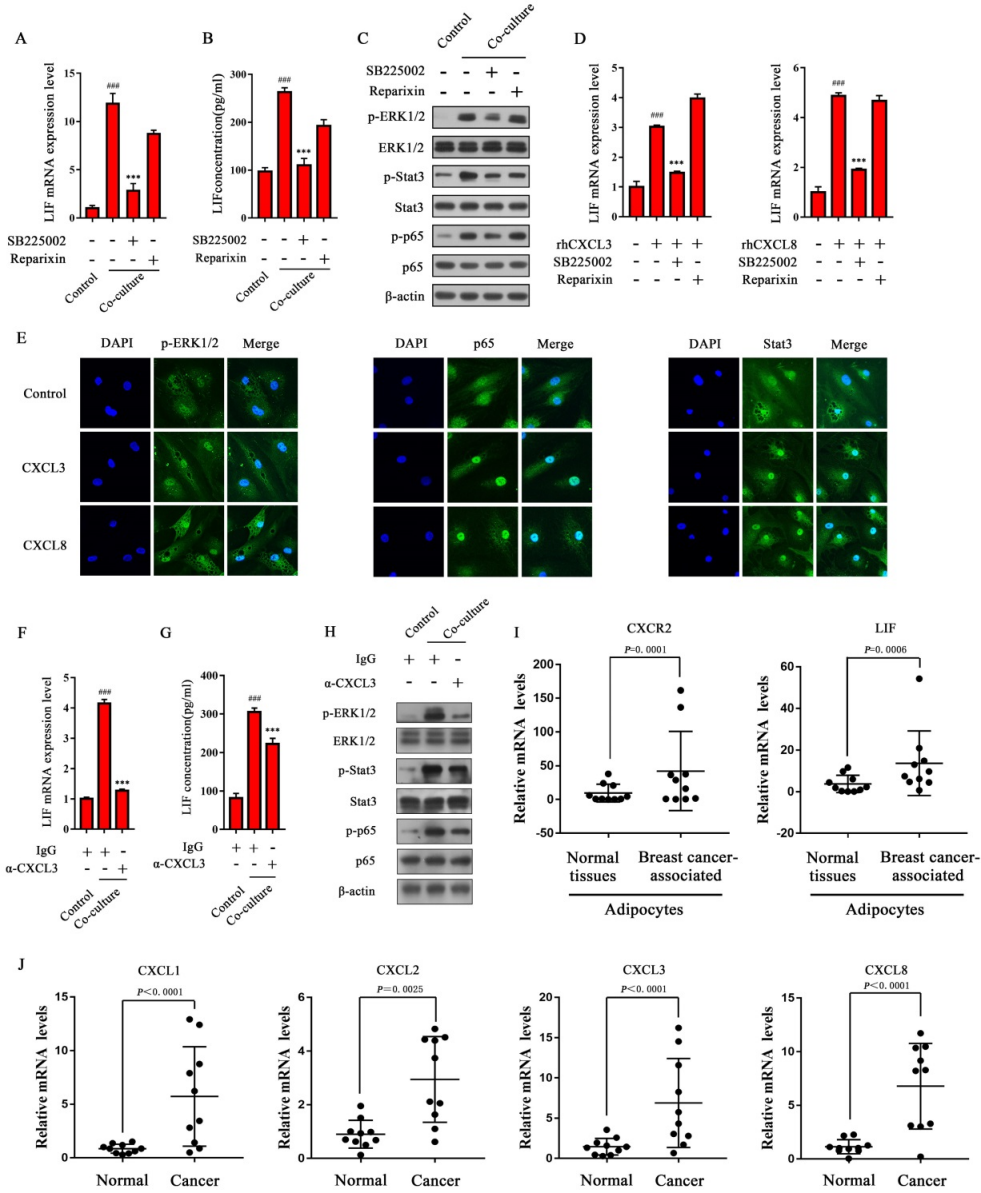
** CXCLs activate the ERK1/2 signaling pathway to up-regulate LIF expression in CAAs.** (**A** and **B**) SB225002 or Reparixin was added to the co-culture system. (**A**) The total RNA of adipocytes was extracted for q-PCR analysis, or (**B**) the CAA culture medium was collected for ELISA analysis. (**C**) The protein expression level of adipocytes was detected by western blot. (**D**) Adipocytes were treated with rhCXCL3 or rhCXCL8 in combination with SB225002 and Reparixin. The LIF mRNA levels in adipocytes of each group were detected by q-PCR. (**E**) Adipocytes were treated with rhCXCL3 and rhCXCL8. The expression and localization of p-ERK1/2, NF-κB p65 and Stat3 in CAAs were observed using a fluorescent confocal microscope, scale bar: 20 μm. (**F** and **G**) α-CXCL3 (5 μg/mL) was added in the co-cultured system. The adipocyte RNA was extracted for q-PCR analysis (**F**), or the CAA culture medium was collected for ELISA analysis (**G**). (**H**) The protein expression level of adipocytes was analyzed by western blot. (**I**) The mRNA level of CXCR2 and LIF in adipocytes next to the normal breast tissue and breast cancer tissue were detected by q-PCR. (**J**) The mRNA level of CXCL1-3 and CXCL8 in normal breast tissue and breast cancer tissue was detected by q-PCR. Typical microscopic fields and blots are shown and quantitative data are presented as the mean ± SD from at least three independent experiments. ^#^*p* < 0.05, ^##^*p* < 0.01, ^###^*p* < 0.001, Co-culture or rhCXCL3/8 VS. Control; *p < 0.05, **p < 0.01, ***p < 0.001, Co-culture + inhibitors/antibody or rhCXCL3/8 + inhibitors VS. Co-culture or rhCXCL3/8.

There are two receptors for CXCL1-3 and CXCL8, namely CXCR1 and CXCR2, of which CXCL1-3 and CXCL8 can bind to CXCR2, and CXCL8 can also bind to CXCR1 to exert biological effects 27, 28. In order to explore whether the receptors CXCR1 and CXCR2 on CAA are involved in the regulation of LIF expression during co-culturing, inhibitors SB225002 (inhibiting the binding of chemokines to CXCR2) and Reparixin (targeting CXCR1 and CXCR2, but the inhibitory effect of CXCR1 is 400 times more potent than that of targeting CXCR2 29) were added to the co-culture system of adipocytes and MDA-MB-231 cells. The results showed that SB225002 significantly down-regulates LIF mRNA level in CAAs, while Reparixin has no significant effect on the level of LIF mRNA in CAAs (Figure 7A). Consistently, the ELISA results showed that the secretion of LIF in CAAs was inhibited by SB225002, while Reparixin did not affect the secretion of LIF in CAAs (Figure 7B), indicating that the CAA surface receptor CXCR2, not CXCR1, is involved in up-regulating the expression of LIF in CAAs. Consistently, SB225002 can reverse the increased phosphorylation of ERK1/2, Stat3, and NF-κB p65 in CAAs, while Reparixin does not significantly inhibit these signals (Figure 7C), indicating that the receptor CXCR2, but not CXCR1, is involved in activating ERK1/2, Stat3, and NF-κB signals to up-regulate LIF expression in CAAs.

### CXCL3 and CXCL8 upregulate the expression of LIF by the CXCR2/ERK1/2 signaling pathway in adipocytes

Combined with RNA-seq analysis, we found that CXCL1-3 and CXCL8 are highly expressed in co-cultured breast cancer cells, and CXCR2 is the critical receptor to activate the ERK1/2 signaling pathway in CAAs. Therefore, the CXCLs/CXCR2 axis may mediate LIF expression in CAAs. To confirm this, adipocytes were treated with rhCXCL3 and rhCXCL8, and the results showed that they up-regulated the expression of LIF mRNA (Figure 7D). Then, we evaluated the effects of rhCXCL3 and rhCXCL8 on the nuclear translocation of Stat3 and p65 in adipocytes by immunofluorescence. The results showed that after treatment with rhCXCL3 and rhCXCL8, ERK1/2 phosphorylation in adipocytes increased, and the expression of p65 and Stat3 in the nucleus were more pronounced (Figure 7E). This shows that CXCL3 and CXCL8 activate ERK1/2, which then stimulates NF-κB p65 and Stat3 to enter the nucleus in adipocytes to initiate LIF expression.

To further verify the effect of breast cancer-derived CXCLs on the expression of LIF in CAAs and its upstream signaling pathway, the neutralizing antibody of CXCL3 (α-CXCL3) was used in the co-culture system to evaluate the expression level of LIF. The results showed that α-CXCL3 could significantly inhibit the level of LIF mRNA in CAAs induced by breast cancer cells (Figure 7F), and the ELISA results showed that the secretion of LIF in CAAs was inhibited by α-CXCL3 (Figure 7G). The phosphorylation of ERK1/2, Stat3, and NF-κB p65 in CAAs was also reversed by α-CXCL3 (Figure 7H). In addition, we detected the mRNA levels of CXCL1-3 and CXCL8 in cancer tissues, and CXCR2 and LIF in adipocytes adjacent to breast cancer of clinical breast cancer specimens. Compared with normal breast tissue, significantly elevated mRNA levels of CXCR2 and LIF in breast cancer-associated adipocytes (Figure 7I), and the expression of CXCL1-3, and CXCL8 in breast cancer tissue were up-regulated (Figure 7J). These results indicate that breast cancer cell-derived CXCLs can activate the ERK1/2 signaling pathway in CAAs and further activate Stat3 and NF-κB to up-regulate LIF expression.

### LIF up-regulates the expression of CXCLs

**Figure 8:**
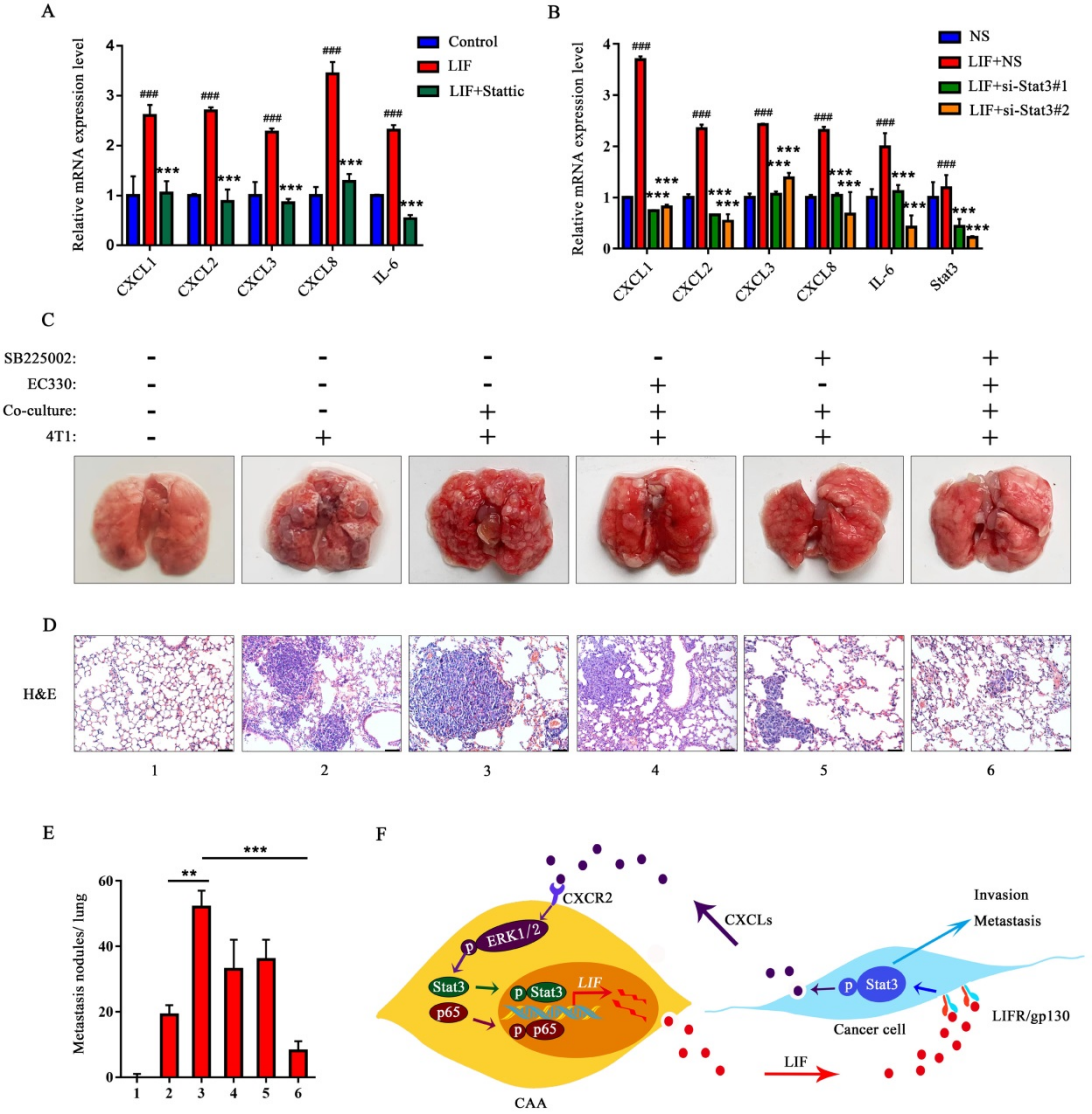
** EC330 and SB225002 reduce lung metastasis of co-cultured 4T1 cells.** (**A**) MDA-MB-231 cells were treated with rhLIF and Stattic. The mRNA level of CXCLs in MDA-MB-231 cells was detected. (**B**) MDA-MB-231 cells transfected with siRNA targeting Stat3 or non-specific (NS) were treated with rhLIF, and the mRNA level of CXCLs was detected. (**C**) Representative lungs harvested at necropsy after tail vein injection with PBS or 4T1 cells previously co-cultured with or without adipocytes and added EC330 and SB225002 alone or in combination. (**D**) Representative images of H&E staining of lungs from mice injected with PBS or 4T1 cells previously co-cultured with or without adipocytes and added EC330 and SB225002 alone or in combination, scale bar: 100 μm. (**E**) Metastasis nodules number obtained from mice injected with PBS or 4T1 cells previously co-cultured with or without adipocytes and added EC330 and SB225002 alone or in combination. (**F**) A model for the bidirection communication between CAA and breast cancer. Quantitative data are presented as the mean ± SD from at least three independent experiments. ^#^*p* < 0.05, ^##^*p* < 0.01, ^###^*p* < 0.001, LIF or LIF + NS VS. Control or NS; *p < 0.05, **p < 0.01, ***p < 0.001, LIF + inhibitors or si-Stat3 + LIF VS. LIF or NS + LIF.

Considering that after co-culturing with adipocytes, the expression and secretion of CXCLs in breast cancer cells increased, and since studies have shown that LIF can induce the expression of CXCL8 to promote the proliferation of pancreatic cancer cells 30, we speculate that the expression of CXCLs may be regulated by the LIF/Stat3 signaling pathway in breast cancer cells. The rhLIF and Stattic were added to MDA-MB-231 cells. The mRNA levels of CXCL1-3, CXCL8, and IL-6 increased in MDA-MB-231 cells treated with LIF, and the mRNA levels of CXCLs and IL-6 were significantly inhibited by Stattic (Figure 8A). Similarly, the regulation of CXCLs by LIF was inhibited after targeted silencing of endogenous Stat3 (Figure 8B). These results indicate that the LIF/Stat3 axis regulate the expression of CXCLs in breast cancer.

### Combination of EC330 and SB225002 attenuates 4T1 lung metastasis by inhibiting intercellular communication between 3T3-L1 and 4T1

Next, we investigated the effect of treatment by the small molecule inhibitors EC330 (a small molecule compound that effectively inhibits the function of LIF in promoting the proliferation and migration of cancer cells 31) and SB225002 (targeting CXCR2) on lung metastasis. First, we induced 3T3-L1 to differentiate into mature adipocytes, and co-cultured these mature adipocytes with 4T1 for 24 h. We detected the upregulation of inflammatory factors IL-6, CXCR2 and LIF, and significant changes in differentiation markers PPAR-γ and C/EBP-α (Supplement Figure 4A). In addition, ERK1/2, Stat3, and p65 signaling were significantly activated in co-cultured 3T3-L1 adipocytes (Supplement Figure 4B). At the same time, Stat3 phosphorylation and CXCLs expression levels increased in 4T1 that had experienced co-culture (Supplement Figure 4C and D). Therefore, we suggest that the interaction with 4T1 resulted in the conversion of 3T3-L1 to CAA.

Each group of 4T1 cells was injected intravenously in mice for tumor metastasis assay. The number and size of metastatic nodules were significantly increased in the co-culture group compared to the group injected with 4T1 alone (Figure 8C). The metastatic nodules in the co-culture group treated with EC330 or SB225002 were slightly reduced, and the reduction was more significant in the co-culture group treated with the combination of EC330 and SB225002 (Figure 8C and E). H&E staining analysis further confirmed the tumor metastasis. As shown in the Figure 8D, more tumor metastases were observed the images of lung section from the co-culture group. Histologic analysis confirmed a mild treatment effect in lung nodules using the EC330 or SB225002 compared to the group injected with 4T1 alone, and a marked reduction of lung nodules in the combined EC330 and SB225002 treatment group (Figure 8D and E). These results suggest that the combination of EC330 and SB225002 enhances the inhibition of CAA-promoted tumor metastasis to the lung. Based on all the above results, we propose a working diagram depicted in Figure 8F. According to this model, the CXCLs and LIF mediated signaling pathways form a malignant positive feedback loop between adipocytes and breast cancer cells to promote breast cancer progression.

## Discussion

Several clinical studies have demonstrated that the invasion of adipose tissue at the tumor invasive front influences lymph nodal metastasis and is linked with poor prognosis 5, 32, which indicates the importance of adipocytes in the progression of breast cancer. In our present study, after co-culturing, the lipid droplets of adipocytes and the expression of terminal differentiation markers PPAR-γ and C/EBP-α were significantly decreased, while the expression of pro-inflammatory cytokines IL-1β, IL-6 and CCL2 increased in adipocytes. These results are consistent with the phenotypic characteristics of CAA 3.

CAA is known to secrete a variety of substances including cytokines, metabolites and exosomes, which may be involved in the malignant transformation of breast cancer cells 10. Our results confirmed that the expression of LIF was significantly increased in CAAs. Usually, the abnormal expression of LIF may be related to the malignant progression of tumors 33, 34. However, the biological function of LIF in the breast cancer microenvironment is unclear. We found that the expression of LIF and its receptor increased in co-cultured adipocytes and breast cancer cells, respectively. After neutralizing the LIF of CAA-CM by using LIF neutralizing antibodies, CAA's promotion of breast cancer cell migration and invasion was inhibited.

To explore how LIF promotes cell migration and invasion in breast cancer, the downstream targets were detected and we found that selective inhibitors or siRNA of Stat3 leads to a decrease in the migration and invasion of breast cancer cells induced by rhLIF or CAAs. LIF was highly expressed in adipocytes adjacent to breast cancer of clinical breast cancer specimens, which was correlated with Stat3 phosphorylation in breast cancer. Our results indicated that LIF plays an important role in CAA-mediated migration and invasion of breast cancer cells by regulating the activation of Stat3.

To determine the molecular mechanism for the high expression of LIF in CAAs, we explored the regulatory of the LIF upstream signaling pathway. Our results show that the ERK1/2 was confirmed to be an upstream activator of transcription factors NF-κB and Stat3 by specific inhibitors, and the ERK1/2 signal up-regulates LIF expression by activating NF-κB and Stat3 and inducing them into the nucleus.

However, it is still unclear how the ERK1/2 signaling pathway is activated in CAAs. Chemokines are involved in many biological processes, such as embryogenesis, angiogenesis and tumor metastasis 27. ELR+CXC chemokines belong to the CXC subgroup of chemokines, including CXCL1, CXCL2, CXCL3, CXCL5, CXCL6, CXCL7, and CXCL8 35, 36. CXCR2 is the main receptor for ELR+CXC chemokines to mediate tumorigenesis and development 37, and high expression of CXCR2 is associated with poor prognosis in cancers 38. Our RNA-seq results showed that the expression of CXCL1, CXCL2, CXCL3, and CXCL8 was significantly increased in breast cancer cells after co-culturing with adipocytes. By targeting the receptors on CAAs with specific inhibitors, CXCR2, but not CXCR1, played an important role in inducing LIF expression and activating the ERK1/2 signaling pathway. The LIF/Stat3 axis induced a massive release of CXCLs from breast cancer cells into their surrounding microenvironment, which is responsible for the uptake of CXCLs by CAA from the microenvironment and the sustained activation of ERK1/2 and its downstream targets, such as NF-κB and Stat3. Importantly, the role of CAA in promoting breast cancer progression has been confirmed *in vivo*, and the combined targeting of LIF and CXCR2 can significantly reduce breast cancer metastasis.

Here, we reveal the significance of paracrine in cytokines acting and mediating the communication between cancer cells and surrounding stromal cells in the tumor microenvironment. Therefore, cancer research should shift its target from the internal pathways of cancer cells to the crosstalk between tumors and their microenvironment 39.

In conclusion, our results clarified that during the communication between CAAs and breast cancer cells, CAA-derived LIF promotes breast cancer cell migration and invasion by activating the Stat3 signal. Importantly, we found that breast cancer-derived CXCL1-3 and CXCL8 bind to the receptor CXCR2 and activate the ERK1/2 signaling pathway to initiate the expression of LIF by activating transcription factors NF-κB and Stat3 in CAAs in paracrine manner. Conversely, LIF up-regulate the expression of CXCLs in breast cancer cells. Therefore, the chemokines CXCLs and the pro-inflammatory factor LIF mediated signaling pathways form a positive feedback loop between adipocytes and breast cancer cells to promote breast cancer progression.This might provide a novel direction for the pathogenesis of breast cancer and a new intervention target for clinical investigations.

## Supplementary Material

Supplementary figures and tables.

## References

[1] Siegel RL, Miller KD, Jemal A (2020). Cancer statistics, 2020. CA Cancer J Clin.

[2] Rybinska I, Agresti R, Trapani A, Tagliabue E, Triulzi T (2020). Adipocytes in Breast Cancer, the Thick and the Thin. Cells.

[3] Dirat B, Bochet L, Dabek M, Daviaud D, Dauvillier S, Majed B (2011). Cancer-associated adipocytes exhibit an activated phenotype and contribute to breast cancer invasion. Cancer research.

[4] Wang YY, Lehuede C, Laurent V, Dirat B, Dauvillier S, Bochet L (2012). Adipose tissue and breast epithelial cells: a dangerous dynamic duo in breast cancer. Cancer Lett.

[5] Yamaguchi J, Ohtani H, Nakamura K, Shimokawa I, Kanematsu T (2008). Prognostic impact of marginal adipose tissue invasion in ductal carcinoma of the breast. Am J Clin Pathol.

[6] Wu Q, Li J, Li Z, Sun S, Zhu S, Wang L (2019). Exosomes from the tumour-adipocyte interplay stimulate beige/brown differentiation and reprogram metabolism in stromal adipocytes to promote tumour progression. J Exp Clin Cancer Res.

[7] Nieman KM, Kenny HA, Penicka CV, Ladanyi A, Buell-Gutbrod R, Zillhardt MR (2011). Adipocytes promote ovarian cancer metastasis and provide energy for rapid tumor growth. Nature medicine.

[8] Choi J, Cha YJ, Koo JS (2018). Adipocyte biology in breast cancer: From silent bystander to active facilitator. Prog Lipid Res.

[9] Rybinska I, Mangano N, Tagliabue E, Triulzi T (2021). Cancer-Associated Adipocytes in Breast Cancer: Causes and Consequences. Int J Mol Sci.

[10] Wu Q, Li B, Li Z, Li J, Sun S, Sun S (2019). Cancer-associated adipocytes: key players in breast cancer progression. J Hematol Oncol.

[11] Liu L, Wu Y, Zhang C, Zhou C, Li Y, Zeng Y (2020). Cancer-associated adipocyte-derived G-CSF promotes breast cancer malignancy via Stat3 signaling. J Mol Cell Biol.

[12] La Camera G, Gelsomino L, Malivindi R, Barone I, Panza S, De Rose D (2021). Adipocyte-derived extracellular vesicles promote breast cancer cell malignancy through HIF-1alpha activity. Cancer Lett.

[13] da Rocha MCO, da Silva PB, Radicchi MA, Andrade BYG, de Oliveira JV, Venus T (2020). Docetaxel-loaded solid lipid nanoparticles prevent tumor growth and lung metastasis of 4T1 murine mammary carcinoma cells. J Nanobiotechnology.

[14] Lapeire L, Hendrix A, Lambein K, Van Bockstal M, Braems G, Van Den Broecke R (2014). Cancer-associated adipose tissue promotes breast cancer progression by paracrine oncostatin M and Jak/STAT3 signaling. Cancer research.

[15] Nicola NA, Babon JJ (2015). Leukemia inhibitory factor (LIF). Cytokine Growth Factor Rev.

[16] Santoni M, Miccini F, Cimadamore A, Piva F, Massari F, Cheng L (2021). An update on investigational therapies that target STAT3 for the treatment of cancer. Expert opinion on investigational drugs.

[17] Sgrignani J, Garofalo M, Matkovic M, Merulla J, Catapano CV, Cavalli A (2018). Structural Biology of STAT3 and Its Implications for Anticancer Therapies Development. International journal of molecular sciences.

[18] Liu YN, Niu S, Chen WY, Zhang Q, Tao Y, Chen WH (2019). Leukemia Inhibitory Factor Promotes Castration-resistant Prostate Cancer and Neuroendocrine Differentiation by Activated ZBTB46. Clin Cancer Res.

[19] Yu H, Yin S, Zhou S, Shao Y, Sun J, Pang X (2018). Magnolin promotes autophagy and cell cycle arrest via blocking LIF/Stat3/Mcl-1 axis in human colorectal cancers. Cell Death Dis.

[20] Chen D, Sun Y, Wei Y, Zhang P, Rezaeian AH, Teruya-Feldstein J (2012). LIFR is a breast cancer metastasis suppressor upstream of the Hippo-YAP pathway and a prognostic marker. Nat Med.

[21] Zhang C, Liu J, Wang J, Hu W, Feng Z (2021). The emerging role of leukemia inhibitory factor in cancer and therapy. Pharmacol Ther.

[22] Schust J, Sperl B, Hollis A, Mayer TU, Berg T (2006). Stattic: a small-molecule inhibitor of STAT3 activation and dimerization. Chem Biol.

[23] Han L, Song S, Niu Y, Meng M, Wang C (2017). Eicosapentaenoic Acid (EPA) Induced Macrophages Activation through GPR120-Mediated Raf-ERK1/2-IKKbeta-NF-kappaB p65 Signaling Pathways. Nutrients.

[24] Lyu JH, Huang B, Park DW, Baek SH (2016). Regulation of PHLDA1 Expression by JAK2-ERK1/2-STAT3 Signaling Pathway. J Cell Biochem.

[25] Zhu N, Gu L, Jia J, Wang X, Wang L, Yang M (2019). Endothelin-1 triggers human peritoneal mesothelial cells' proliferation via ERK1/2-Ets-1 signaling pathway and contributes to endothelial cell angiogenesis. J Cell Biochem.

[26] Hodge C, Liao J, Stofega M, Guan K, Carter-Su C, Schwartz J (1998). Growth hormone stimulates phosphorylation and activation of elk-1 and expression of c-fos, egr-1, and junB through activation of extracellular signal-regulated kinases 1 and 2. J Biol Chem.

[27] Hughes CE, Nibbs RJB (2018). A guide to chemokines and their receptors. FEBS J.

[28] Korbecki J, Kojder K, Kapczuk P, Kupnicka P, Gawronska-Szklarz B, Gutowska I (2021). The Effect of Hypoxia on the Expression of CXC Chemokines and CXC Chemokine Receptors-A Review of Literature. Int J Mol Sci.

[29] Bertini R, Barcelos LS, Beccari AR, Cavalieri B, Moriconi A, Bizzarri C (2012). Receptor binding mode and pharmacological characterization of a potent and selective dual CXCR1/CXCR2 non-competitive allosteric inhibitor. Br J Pharmacol.

[30] Kamohara H, Takahashi M, Ishiko T, Ogawa M, Baba H (2007). Induction of interleukin-8 (CXCL-8) by tumor necrosis factor-alpha and leukemia inhibitory factor in pancreatic carcinoma cells: Impact of CXCL-8 as an autocrine growth factor. Int J Oncol.

[31] Yue X, Wu F, Wang J, Kim K, Santhamma B, Dileep KV (2020). EC330, a small-molecule compound, is a potential novel inhibitor of LIF signaling. J Mol Cell Biol.

[32] Kimijima I, Ohtake T, Sagara H, Watanabe T, Takenoshita S (2000). Scattered fat invasion: an indicator for poor prognosis in premenopausal, and for positive estrogen receptor in postmenopausal breast cancer patients. Oncology.

[33] Liu SC, Tsang NM, Chiang WC, Chang KP, Hsueh C, Liang Y (2013). Leukemia inhibitory factor promotes nasopharyngeal carcinoma progression and radioresistance. J Clin Invest.

[34] Wu L, Yu H, Zhao Y, Zhang C, Wang J, Yue X (2015). HIF-2alpha mediates hypoxia-induced LIF expression in human colorectal cancer cells. Oncotarget.

[35] Bikfalvi A, Billottet C (2020). The CC and CXC chemokines: major regulators of tumor progression and the tumor microenvironment. American journal of physiology Cell physiology.

[36] Strieter RM, Polverini PJ, Kunkel SL, Arenberg DA, Burdick MD, Kasper J (1995). The functional role of the ELR motif in CXC chemokine-mediated angiogenesis. The Journal of biological chemistry.

[37] Cheng Y, Ma XL, Wei YQ, Wei XW (2019). Potential roles and targeted therapy of the CXCLs/CXCR2 axis in cancer and inflammatory diseases. Biochimica et biophysica acta Reviews on cancer.

[38] Saintigny P, Massarelli E, Lin S, Ahn YH, Chen Y, Goswami S (2013). CXCR2 expression in tumor cells is a poor prognostic factor and promotes invasion and metastasis in lung adenocarcinoma. Cancer Res.

[39] Song YC, Lee SE, Jin Y, Park HW, Chun KH, Lee HW (2020). Classifying the Linkage between Adipose Tissue Inflammation and Tumor Growth through Cancer-Associated Adipocytes. Mol Cells.

