# Supplementary material for: Cancer-associated adipocytes promote the invasion and metastasis in breast cancer through LIF/CXCLs positive feedback loop
Source: Int J Biol Sci. 2022 Jan 16;18(4):1363–80. doi: 10.7150/ijbs.65227 (PMC8898379; doi:10.7150/ijbs.65227)
Supplement: Supplementary file 1 — Supplementary figures and tables. [file ijbsv18p1363s1.pdf]

Cancer-associated adipocytes promote the invasion and metastasis in breast cancer through LIF/CXCLs positive feedback loop

Chong Zhou[1,7], Xi He[1], Chang Tong[3], Honghui Li[1], Caifeng Xie[1], Yudong Wu[2], Lieliang Wang[2], Xiaohua Yan[1], Daya Luo[1], Yunpeng Tang[4], Zhongman Cheng[5], Xiangyang Xiong[1,6]*

List of Supplementary materials

Supplement table 1: Concentration and target of inhibitors

Supplement table 2: The sequences of primers for q-PCR

Supplement table 3: Information on 10 patients with breast cancer

Supplement figure 1: Formation of CAA

Supplement figure 2: CAA-derived LIF promotes breast cancer cell migration and invasion

Supplement figure 3: Stattic inhibits the migration and invasion and Stat3 phosphorylation of BT549 cells induced by rhLIF and CAA-CM

Supplement figure 4: The validation of a co-culture system for 3T3-L1 and 4T1 cells

Supplementary Table 1 Concentration and target of inhibitors.

Names	Targets	Concentration (μM)
Stattic	Stat3	10
LY3214996	ERK1/2	2
PD98059	MEK1/2, ERK1/2	10
SB 203580	p38, p38β2	10
LY294002	PI3Kα, PI3Kδ, PI3Kβ, CK2, CK2α2, DNA-PK	5
JSH-23	NF-κB	10
BAY 11-7082	NF-κB, USP7, USP21	5
T-5224	AP-1, c-fos	10
TK216	ETS	1
TAT-DEF-Elk-1	Elk-1	20
SB225002	CXCR2	0.1
Reparixin	CXCR1, CXCR2 (the efficiency of inhibiting CXCR1 activity is 400 times that of CXCR2)	0.1
SB-505124	TGF-β Receptor type I receptor	5
EC330	LIF	1

Supplementary Table 2 The sequences of primers for q-PCR.

Species	Genes	q-PCR Primers (5'-3')
	GAPDH	F-CATCTTCCAGGAGCGAGACC
		R-CTCGTGGTTCACACCCATCA
	S18	F-ATCACCATTATGCAGAATCCACG
		R-GACCTGGCTGTATTTTCCATCC
	LIF	F- CCAACGTGACGGACTTCCC
		R- TACACGACTATGCGGTACAGC
	LIFR	F- AGATATGCCCTTGGAATGTGC
		R- TCCACTCTTCGAGACCAGAAAA
Human	gp130	F- GTGAGTGGGATGGTGGAAGG
		R- CAAACTTGTGTGTTGCCCATTC
	CCL2	F- CAGCCAGATGCAATCAATGCC
		R- TGGAATCCTGAACCCACTTCT
	CCL20	F- CAGCACTCCCAAAGAACTGG
		R- CACTGACATCAAAGCAGCCA
	IL-1β	F- AGCTACGAATCTCCGACCAC
		R- CGTTATCCCATGTGTCGAAGAA
	IL-6	F- CCTGAACCTTCCAAAGATGGC
		R- TTCACCAGGCAAGTCTCCTCA
	IL-11	F- GGACCACAACCTGGATTCCCTG
		R- AGTAGGTCCGCTCGCAGCCTT

	CXCL1	F- TCCTGCATCCCCCATAGTTA
		R- CTTCAGGAACAGCCACCAGT
	CXCL2	F- GAAAGCTTGTCTCAACCCCG
		R- TCTCCTAAGTGATGCTCAAACA
	CXCL3	F- GAGCACCAACTGACAGGAGA
		R- AGTCCTTTCCAGCTGTCCCTA
	CXCL8	F- TTTTGCCAAGGAGTGCTAAAGA
		R- AACCCTCTGCACCCAGTTTTC
	CXCR2	F- AGCACTCATCCCAGAATCACTA
		R- GGGCTTTTCACCTGTAGGACA
	CSF2	F- GCGTCTCCTGAACCTGAGTA
		R-ATGCTGAAGCTCACACCCTT
	CSF3	F- GAGCAAGTGAGGAAGATCCAG
		R- CAGCTTGTAGGTGGCACACA
	STC-1	F- GTGGCGGCTCAAAACTCAG
		R- GTGGAGCACCTCCGAATGG
	PTX3	F- AGGCTTGAGTCTTTTAGTGCC
		R- ATGGATTCCTCTTTGTGCCATAG
	PPAR-γ	F- ACCAAAGTGCAATCAAAGTGGA
		R- ATGAGGGAGTTGGAAGGCTCT
	C/EBP-α	F- TATAGGCTGGGCTTCCCCTT
		R- AGCTTTCTGGTGTGACTCGG
	HSL	F- AGGAGCCAGCATTGAGACAAA
		R- CGCAGGTGTTGATTCAGCTTC
	PREF1	F- CTTTCGGCCACAGCACCTAT
		R- TGTCATCCTCGCAGAATCCAT
	FABP4	F- ACTGGGCCAGGAATTTGACG
		R- CTCGTGGAAGTGACGCCTT
Mouse	CXCL1	F- TGGCTGGGATTCACCTCAAG
		R- CCGTTACTTGGGGACACCTT
	CXCL2	F- GCTGTCCCTCAACGGAAGAA
		R- CGAGGCACATCAGGTACGAT
	CXCL3	F- CCTACCAAGGGTTGATTTTGAGAC
		R- GAGTGGCTATGACTTCTGTCTGG
	IL-6	F- AGTCCTTCCTACCCCAATTTCC
		R- TGGTCTTGGTCCTTAGCCAC
	LIF	F- CCCCTGTAAATGCCACCTGT
		R- CTTCTCTGTCCCGTTGCCAT
	CXCR2	F- CACAAACAGCGTCGTAGAACT
		R- ACCAAGGAGTTCCCCACAAG
	PPAR-γ	F- CTCCAAGAATACCAAAGTGCGA
		R- GCCTGATGCTTTATCCCCACA
	C/EBP-α	F- CAAGAACAGCAACGAGTACCG
		R- GTCACTGGTCAACTCCAGCAC

Supplementary Table 3 Information on 10 patients with breast cancer.

Number	Age	Height(m)	Weight(kg)	BMI	Histology	Stage
**1**	69	148	50	22.83	IDC	3A
**2**	54	159	53	20.96	IDC	2B
**3**	57	154	55	23.19	IDC	2B
**4**	61	155	56	23.31	IDC	3C
**5**	56	156	60	24.65	IDC	2B
**6**	33	165	47	17.26	IDC	2A
**7**	52	158	53	21.23	IDC	2B
**8**	58	156	52	21.37	IDC	3A
**9**	51	160	65	25.39	IDC	3A
**10**	77	145	52	24.73	IDC	3A

Note: IDC, invasion ductal carcinoma

Supplementary figure

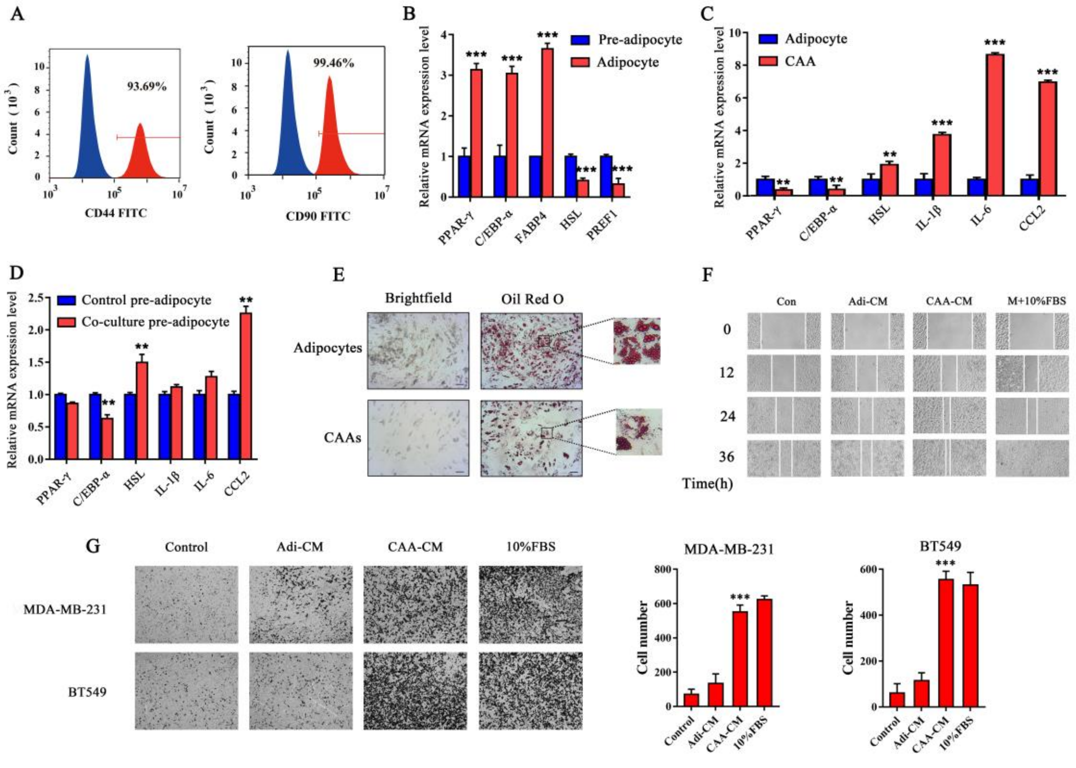

Supplement figure 1. Formation of CAA. (**A**) The CD44 and CD90 expressions of the third generation pre-adipocytes were analyzed by flow cytometry. (**B**) The mRNA expression of differentiation markers in pre-adipocytes and mature adipocytes was detected by q-PCR. (**C and D**) The mRNA expression of differentiation markers and inflammatory factors in adipocytes and pre-adipocytes was detected by q-PCR. (**E**) Oil red O staining showed the morphology of lipid droplets in adipocytes cultured alone or co-cultured adipocytes (CAA), scale bar: 200 μm. (**F and

G) MDA-MB-231 cells were stimulated by DMEM (negative control group), Adi-CM, CAA-CM and DMEM containing 10% FBS (positive control group), and the cell migration (**F**) and invasion (**G**) were observed by phase contrast microscope. Typical microscopic fields are shown and quantitative data are presented as mean ± SD from at least three independent experiments. **p* < 0.05, ***p* < 0.01, ****p* < 0.001.

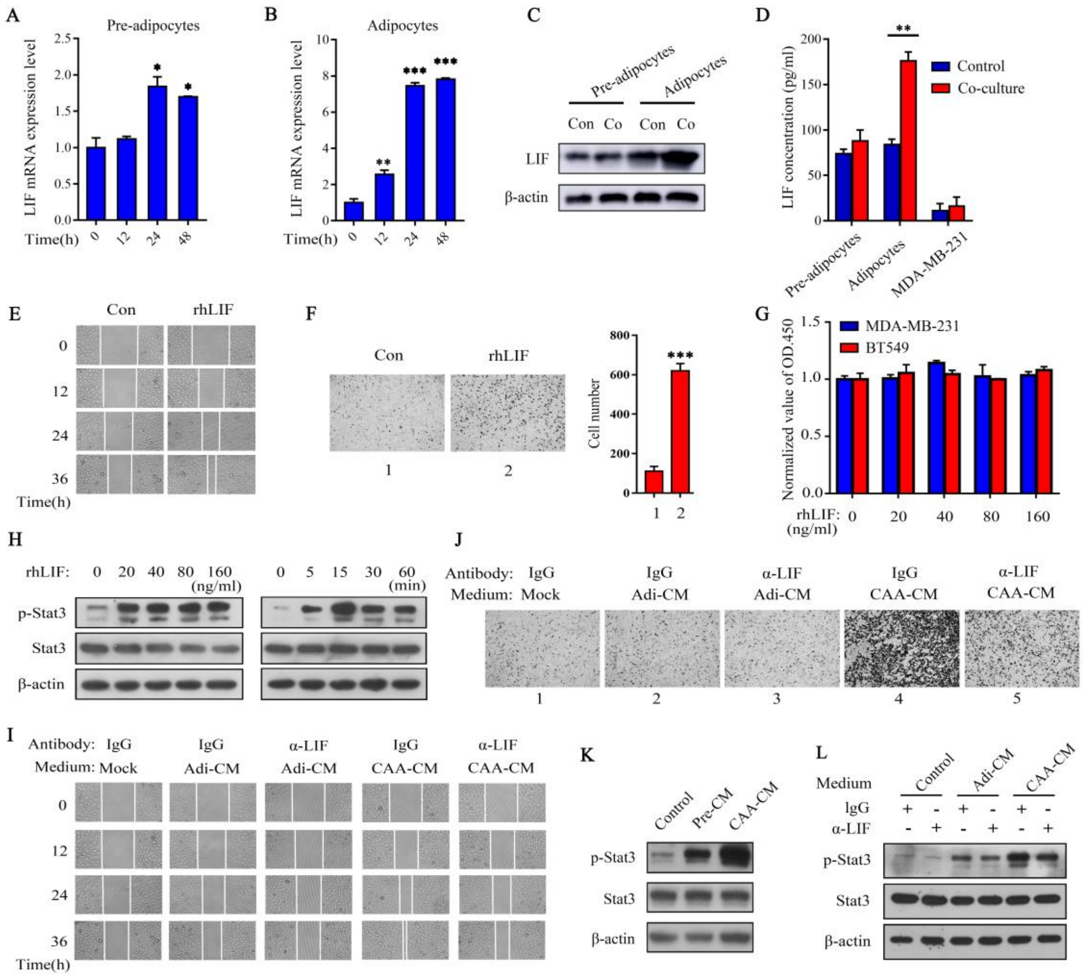

Supplement figure 2. CAA-derived LIF promotes breast cancer cell migration and invasion. (**A and B**) The mRNA expression of LIF in co-cultured pre-adipocytes (**A**) and mature adipocytes (**B**) at different time was detected by q-PCR. (**C**) The protein expression of LIF in co-cultured pre-adipocytes and mature adipocytes was analyzed by western blot. (**D**) The protein secretion of LIF in co-cultured pre-adipocytes, mature adipocytes and MDA-MB-231 cells was analyzed by ELISA. (**E and F**) BT549 cells were treated with rhLIF and subjected to migration (**E**) and Transwell Matrigel invasion assays (**F**). (**G**) MDA-MD-231 and BT549 cells were treated with rhLIF (0, 20, 40, 80, 160 ng/ml) for 48 h. DMEM containing 0.2% FBS was used as a control, and the effect of rhLIF on breast cancer cell proliferation was analyzed by MTT assay. (**H**) MDA-MD-231 cells were treated with rhLIF（0, 20, 40, 80, and 160 ng/mL）for 15 min and with rhLIF for different times (0, 5, 15, 30, 60 min). Stat3 phosphorylation was analyzed by western

blot. (**I and J**) BT549 cells cultured in Adi-CM or CAA-CM or DMEM were treated with LIF-neutralizing antibody or IgG, and the cell migration (**I**) and invasion (**J**) were observed by phase contrast microscope. Typical microscopic fields and blots are shown, and quantitative data are presented as the mean ± SD from at least three independent experiments. (**K**) BT549 cells were treated with DMEM or culture media of co-cultured pre-adipocytes or culture media of CAA for 15 min, and Stat3 phosphorylation was analyzed by western blot. (**L**) BT549 cells were treated with different culture media and LIF-neutralizing antibody or IgG for 15 min, and Stat3 phosphorylation was analyzed by western blot. *p < 0.05, **p < 0.01, ***p < 0.001.

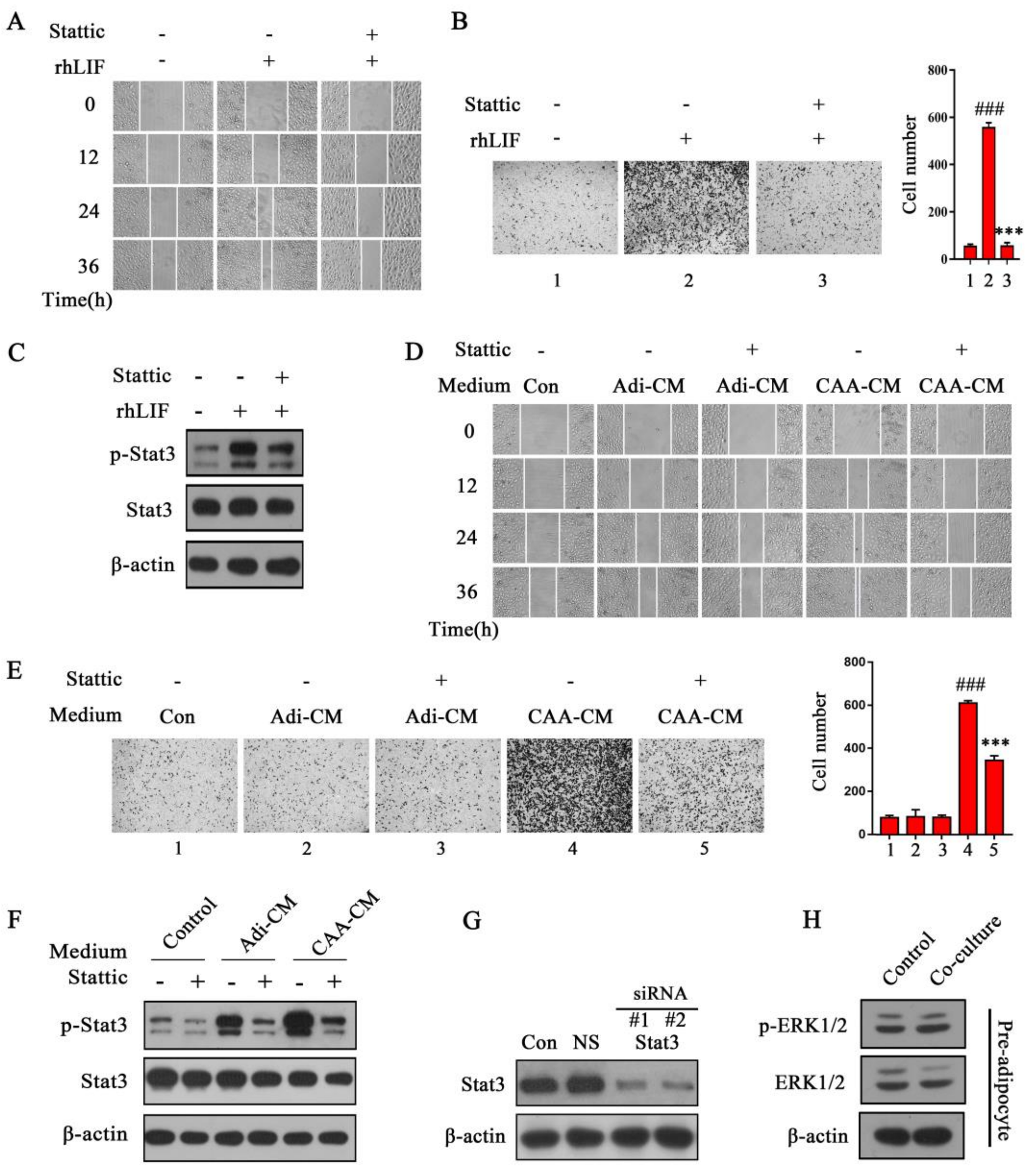

Supplement figure 3. Stattic inhibits the migration and invasion and Stat3 phosphorylation of BT549 cells induced by rhLIF and CAA-CM. (**A and B**) BT549 cells were stimulated with rhLIF or DMEM and Stattic and subjected to migration (**A**) and Transwell Matrigel invasion (**B**) assays. (**C**) BT549 cells were stimulated by rhLIF combined with Stattic for 15 min, the protein

expression level was analyzed by western blot. (**D** and **E**) BT549 cells were stimulated by DMEM, Adi-CM or CAA-CM combined with Stattic and subjected to migration (**D**) and Transwell Matrigel invasion (**E**) assays. (**F**) BT549 cells were treated with DMEM or different adipocyte culture medium with Stattic for 15 min, and the protein expression levels were analyzed by western blot. (**G**) Western blot was performed on MDA-MB-231 cells transfected with non-specific (NS) or Stat3 siRNA and wild-type MDA-MB-231 cells (Con). (**H**) The p-ERK1/2 expression level in pre-adipocytes was analyzed by western blot. Typical microscopic fields and blots are shown and quantitative data are presented as mean ± SD from at least three independent experiments. $^{\#}p < 0.05$, $^{\#\#}p < 0.01$, $^{\#\#\#}p < 0.001$, rhLIF or CAA-CM VS. Con; $^{*}p < 0.05$, $^{**}p < 0.01$, $^{***}p < 0.001$, rhLIF + Stattic VS. rhLIF or CAA-CM + Stattic VS. CAA-CM.

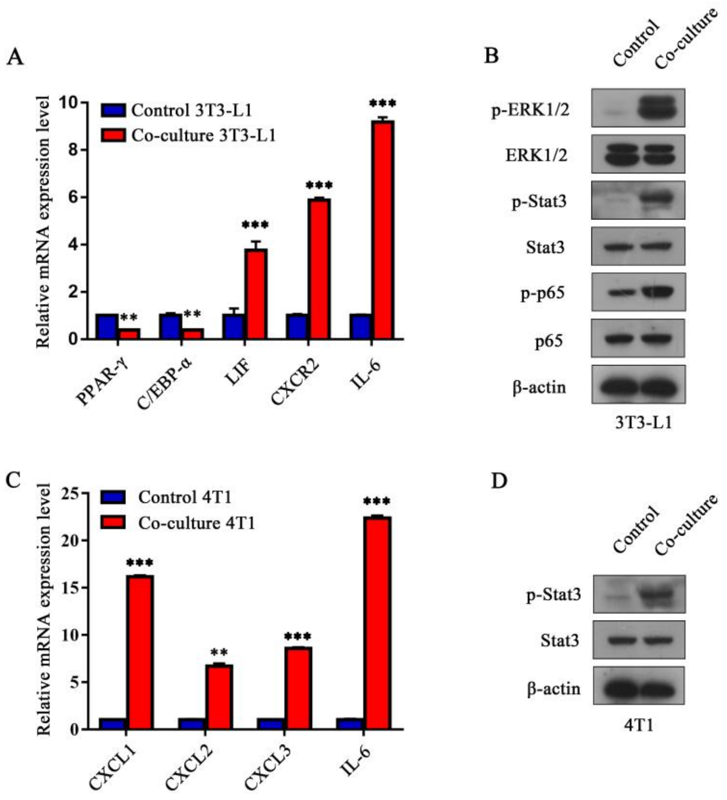

Supplement figure 4. The validation of a co-culture system for 3T3-L1 and 4T1 cells. (**A**) The mRNA expression of differentiation marker genes, LIF, CXCR2 and IL-6 in control and co-cultured 3T3-L1 was detected by q-PCR. (**B**) The protein expression levels of signaling pathway in control and co-cultured 3T3-L1 were analyzed by western blot. (**C**) The mRNA expression of CXCL1-3 and IL-6 in control and co-cultured 3T3-L1 was detected by q-PCR. (**D**) The protein expression level of p-Stat3 in control and co-cultured 4T1 cells was analyzed by western blot. The quantitative data are presented as mean ± SD from at least three independent experiments. $^{*}p < 0.05$, $^{**}p < 0.01$, $^{***}p < 0.001$.
